# Directional Ion Transport Enabled by Self‐Luminous Framework for High‐Performance Quasi‐Solid‐State Lithium Metal Batteries

**DOI:** 10.1002/advs.202205108

**Published:** 2022-12-11

**Authors:** Siyang Ye, Fei Tian, Kaiyuan Shi, Danni Lei, Chengxin Wang

**Affiliations:** ^1^ State Key Laboratory of Optoelectronic Materials and Technologies School of Materials Science and Engineering Sun Yat‐sen (Zhongshan) University Guangzhou 510275 P. R. China

**Keywords:** aluminum ethoxide, fluorescent nanowires, lithium metal batteries

## Abstract

Composite gel polymer electrolyte (CGPE), derived from ceramic fillers has emerged as one of the most promising candidates to improve the safety and cycling stability of lithium metal batteries. However, the poor interface compatibility between the ceramic phase and polymer phase in CGPE severely deteriorates lithium‐ion pathways and cell performances. In this work, a fluorescent ceramic nanowire network that can palliate the energy barrier of photoinitiators and contribute to preferential nucleation and growth of polymer monomers is developed, thus inducing polymer segment orderly arrangement and tightly combination. A proof‐of‐concept study lies on fabrications of poly(ethylene oxide) closely coating on the ceramic nanowires, thus dividing the matrix into mesh units that contribute to directional lithium‐ion flux and dendrite‐free deposition on the metallic anode. The CGPE, based on the state‐of‐the‐art self‐luminous framework, facilitates high‐performance quasi‐solid‐state Li||LiFePO_4_ cell, registering a high capacity of 143.3 mAh g^−1^ after 120 cycles at a mass loading of 12 mg cm^−2^. X‐ray computed tomography provides an insight into the relationship between directional lithium‐ion diffusion and lithium deposition behavior over the electrochemical processes. The results open a door to improve the electrochemical performances of composite electrolytes in various applications.

## Graphical abstract

Luminescent ceramic nanowire network is used as the internal light source to reduce the decomposition energy barrier of photoinitiators, leading to a tight coating of poly(ethylene oxide) on the ceramic nanowires, thus dividing the matrix into mesh units that contribute to directional lithium‐ion flux and dendrite‐free deposition on the metallic anode. The composite gel polymer electrolyte facilitates high‐performance Li||LiFePO_4_ cells.

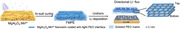

## 1 Introduction

Lithium (Li) metal is considered to be the holy grail anode for lithium rechargeable batteries due to its highest theoretical specific capacity and the lowest electrochemical potential.^[^ ^1^, ^2^ ^]^ However, catastrophic safety problems of thermal runaway and explosion, triggered by Li dendrites penetration and serious side reactions, impede the promise of the application of lithium metal batteries (LMBs).^[^ ^3^, ^4^ ^]^ Composite gel polymer electrolyte (CGPE), derived from ceramic fillers, is considered as an effective strategy to alleviate those issues.^[^ ^5^, ^6^ ^]^ The ceramics in CGPE provide mechanical stiffness to block dendrites while combining advantages of polymers such as flexibility, elasticity, and adhesion.^[^ ^7^, ^8^, ^9^ ^]^ The CGPE materials demonstrate improved stability, outstanding safety record, and excellent electrochemical performance compared to the conventional liquid electrolyte (LE). However, the poor compatibility and non‐conformal coverage between the ceramic phase and polymer phase remain as challenges due to the differences in structure and properties.^[^ ^10^, ^11^, ^12^, ^13^, ^14^, ^15^ ^]^ It has been demonstrated that defects and interfaces within the inhomogeneous CGPE lead to slow and uneven lithium‐ion (Li^+^) migration, and thus affect the formation and growth of Li dendrites.^[^ ^16^, ^17^ ^]^ Therefore, it is critical to develop an efficient method for improving the interfacial compatibility to achieve directional and homogeneous Li^+^ flux to prevent dendrites penetration.^[^ ^18^ ^]^

In recent years, a series of significant studies have been performed to enhance interfacial compatibility.^[^ ^18^, ^19^, ^20^, ^21^, ^22^ ^]^ Forming chemical bonds is an effective way to enhance interfacial compatibility within CGPE. Utilize contaminants on Li_6.75_La_3_Zr_1.75_Ta_0.25_O_12_ (LLZTO) surfaces as reaction initiators to induce a ring‐opening reaction of ethylene carbonate, which further emerged as molecular crosslinkers between LLZTO and poly(ethylene oxide) (PEO).^[^ ^21^ ^]^ However, those approaches are only applicable to garnet pellets. Silane was used as a bridge‐builder to realize the chemical bonding interaction between ceramic fillers and polymer matrix, but the preparation process and Li^+^ transport mechanism become complicated.^[^ ^20^ ^]^ More importantly, the reported composite electrolytes cannot compete with conventional LEs to achieve LMBs with high load (>12 mg cm^−2^) and long cycle life, which is the greatest obstacle to their commercial application. The most fundamental reason is that polymers cannot tightly encapsulate ceramics depending on organic small‐molecule bonding. The organic groups suspended on the surface of the inorganic fillers react with the LE, constantly consuming the LE and causing the battery to suddenly die. What's more, Li^+^ transport mainly in the unconfined amorphous region of the polymer,^[^ ^23^ ^]^ resulting in asymmetrical and low Li^+^ flux at the interface between CGPE and electrode, and thus unable to realize homogeneous Li deposition. Therefore, developing a universal and facial strategy to construct dense and stable interfaces between ceramic fillers and polymer matrix is critical to realize high‐performance solid‐state LMBs.

Herein, we develop a completely new strategy to produce a CGPE with dense and uniform interfaces between 3D inert ceramic fillers and Li^+^ conductor PEO matrix. During the ultraviolet (UV)‐curing process, the red phosphor nanowires MgAl_2_O_4_:Mn^4+^ act as internal light sources to reduce the decomposition energy barrier of the photoinitiators and contribute to preferential nucleation and growth of polymer monomers, which induces polymer segment orderly arrangement and tightly combination. Thus, a dense PEO layer is tightly wrapped on the surface of MgAl_2_O_4_:Mn^4+^, dividing the PEO matrix into mesh units that provide directional Li^+^ conduction pathways.^[^ ^24^, ^25^ ^]^ Consequently, the CGPE presents a high ionic conductivity of 5.66×10^−4^ S cm^−1^ at 25 °C and high voltage stability up to 5.20 V. In addition, an excellent cycling performance (90% capacity retention after 500 cycles at 144.1 mA g^−1^) is achieved in the Li||LiFePO_4_ (LFP) cell with fluorescence MgAl_2_O_4_:Mn^4+^/PEO electrolyte (FMPE). Furthermore, by increasing the mass loading of the LFP cathode to 12 mg cm^−2^, the cell also demonstrates a high capacity retention of 88% after 120 cycles. These findings open a promising avenue to effectively stabilize Li metal and greatly promote the commercialization of all‐solid‐state LMBs.

## 2 Results and Discussion

### 2.1 Reaction Mechanism in CGPEs

Free‐radical chain‐growth is a typical polymerization mechanism, consisting of three steps: initiation (radicals forming), propagation (products developing), and termination (reactions ending).^[^ ^26^, ^27^ ^]^ The initiation which requires the highest activation energy is the rate‐determining step. The process of initiation includes two steps (step (1) and step (2)) shown below:

(1) $$I\overset{k_{d}}{\to }2R•$$

(2) $$R•+M\overset{k_{i}}{\to }RM•$$

Where *M* and *I* represent the concentrations of monomer and initiator, respectively. *R*• and *M*• are the concentrations of primary and monomer radicals, respectively. *k*_d_ and *k*_i_ stand for the rate constants of initiator decomposition and chain initiation, respectively. *k*_d_ is much smaller than *k*_i_, thus step (1) is the rate‐determining step. The factors that affect *k*_d_ can be derived from Equation (3).^[^ ^28^ ^]^

(3) $$\mathrm{In}\;k_{d}=-\frac{E_{d}}{\mathrm{RT}}+\mathrm{In}\;A_{d}$$

Where *E*_d_ stands for the activation energy of initiator decomposition; *A*_d_ is the collision frequency factor; and *T* is the absolute temperature. Therefore, when the temperature is constant, *k*_d_ increases with decreasing *E*_d_. *k*_d_ can be calculated by using Equation (4).^[^ ^28^, ^29^ ^]^

(4) $$\ln\;\frac{{\left[I\right]}_{0}}{\left[I\right]}=k_{d}\;t$$

Where [*I*]_0_ and [*I*] represent the initial and residual concentrations of the initiator, respectively, and *t* stands for the irradiation time.

**Figure 1:**
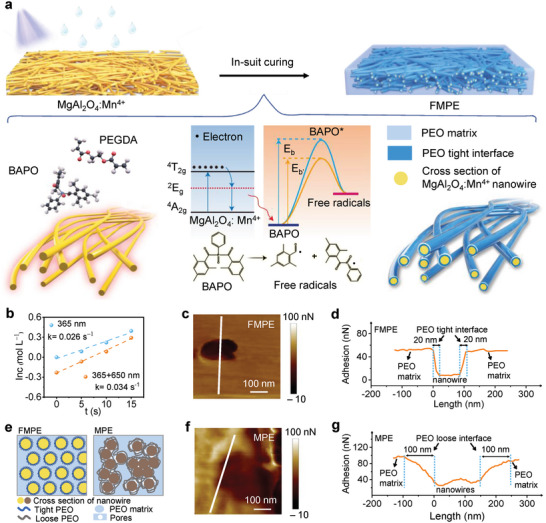
Synthesis mechanism and cross‐sectional AFM characterization of the prepared membranes. a) Schematic showing the synthetic procedures of FMPE. b) Photobleaching curves of BAPO. c) Cross‐sectional AFM adhesion image and d) adhesion‐width curve of FMPE. e) Schematic showing the cross‐sectional of FMPE and MPE. f) Cross‐sectional AFM adhesion image and g) adhesion‐width curve of MPE.

Based on the theory above mentioned, in our work, a fluorescent material was employed to decrease the activation energy of initiator decomposition and contribute to preferential nucleate and growth of polymer monomers, which induces polymer segment orderly arrangement and tightly combination. The photoinitiation mechanism is elucidated in **Figure** **1**a. Under 365 nm UV‐curing, MgAl_2_O_4_:Mn^4+^ nanowires can emit 650 nm red light from the Mn^4+ 2^ *E*_g_ →^4^ *A*_2g_ transition (Figure S1, Supporting Information),^[^ ^30^, ^31^ ^]^ which can be absorbed by the photoinitiators phenylbis(2,4,6‐trimethylbenzoyl) phosphine oxide (BAPO) (Figure S2, Supporting Information). Time‐dependent UV–vis measurements were performed to study the photolysis kinetics of BAPO (Figure S3, Supporting Information) and the calculated *k*_d_ of BAPO under 365 and 650 nm simultaneous irradiation is 0.034 s^−1^, which is higher than that under single‐wavelength 365 nm irradiation (0.026 s^−1^) (Figure 1b). Consequently, *E*_d_ decreases under simultaneous 365 and 650 nm irradiation. Although the energy that BAPO accepts from 650 nm irradiation is insufficient to directly cleave it into free radicals, it can move to an intermediate energy level that is higher than the ground level, and thus effectively decrease *E*_d_. Therefore, the BAPO around MgAl_2_O_4_:Mn^4+^ nanowires has a lower *E*_d_ than the bulk phase, which means that it can break up into free radicals more easily under 365 nm UV light and 650 nm MgAl_2_O_4_:Mn^4+^ emission, and thus monomer free radicals preferentially nucleate and grow on MgAl_2_O_4_:Mn^4+^ nanowires in order to induce chain propagation.^[^ ^32^ ^]^ Thus, a dense PEO layer is tightly wrapped on the surface of MgAl_2_O_4_:Mn^4+^, dividing the PEO matrix into mesh units that provide directional Li^+^ conduction pathways. For comparison, a MgAl_6_O_10_ membrane without 650 nm emission (Figure S4, Supporting Information) was composited to fabricate MgAl_6_O_10_/PEO electrolyte (MPE) using a similar method.

An atomic force microscope (AFM) is widely used to characterize the interfacial properties of composite materials. In our work, the interface between the nanowires and PEO matrix was characterized by measuring the adhesion of cross‐section membranes. The nanowire could be observed clearly in the cross‐sectional AFM height images of FMPE and MPE (Figure S5, Supporting Information), which ensures the line selected in the adhesive images passes through the PEO matrix‐interface‐nanowire. The adhesion image of FMPE membrane processed by NanoScope Analysis software is shown in Figure 1c. The nanowires have a high modulus and low adhesion, while the PEO polymer is inverse with a low modulus and high adhesion. Between them, there exists a tight PEO region that has different properties from the nanowires and PEO matrix. The corresponding line profile of adhesion changes along the PEO matrix‐interface‐nanowire is shown in Figure 1d. We can observe a steep gradient with a slope of 1.04 to 3.26 in the FMPE membrane, and the thickness of the interface between MgAl_2_O_4_:Mn^4+^ nanowires and PEO ranges from 20 to 24 nm, obtained from four random areas (Figure S6, Supporting Information), which indicates a tightly bound PEO layer is formed on the surface of MgAl_2_O_4_:Mn^4+^ nanowires.^[^ ^33^ ^]^ This tight interface region divides the PEO matrix into mesh units (Figure 1e) that contribute to directional Li^+^ conduction pathways and provide uniform Li^+^ flux above Li anode. In contrast, the thickness of the interface between MgAl_6_O_10_ nanowires and the PEO matrix ranges from 100 to 129 nm, with a gentle gradient slope of 0.270 to 0.550 in the MPE membrane (Figure 1f,g and Figure S7, Supporting Information). This wide interface accounts for the weak conglutination between the PEO matrix and MgAl_6_O_10_ nanowires.^[^ ^34^ ^]^ The wide and loose interface allows nanowires to cluster together, leaving the PEO matrix unconfined (Figure 1e), which may result in chaotic Li^+^ flux and the formation of Li dendrites.

### 2.2 Synthesis and Characterization of the MgAl_2_O_4_:Mn^4+^ 3D Interconnected Nanowire Network

The synthesis of the MgAl_2_O_4_:Mn^4+^ 3D interconnected nanowire network generally involved four steps. As illustrated in Figure S8, Supporting Information, the first step was the synthesis of an Al(EtO)_3_ 3D interconnected nanowire membrane based on the method that we have discovered.^[^ ^35^ ^]^ Subsequently, the membrane was heat‐treated at 400 °C to stabilize its construction. Then, the membrane was soaked in a 1.1 mol Mg(CH_3_COO)_2_ solution and heat‐treated at 1200 °C to attain the MgAl_2_O_4_ membrane. Finally, Mn^4+^ was doped in the MgAl_2_O_4_ matrix to obtain the MgAl_2_O_4_:Mn^4+^ 3D interconnected nanowire membrane through a similar third step except for changing the Mg(CH_3_COO)_2_ solution to 0.011 mol Mn (CH_3_COO)_2_ solution. For comparison, a MgAl_6_O_10_ nanowire membrane was fabricated using a similar method. The difference was that the concentration of Mg(CH_3_COO)_2_ solution in the second step was 0.80 mol, and the calcination temperature was 800 °C.

**Figure 2:**
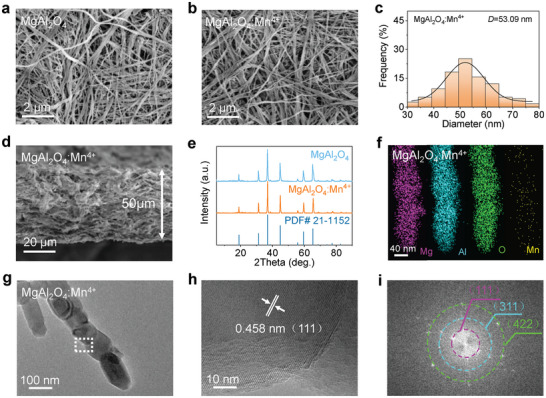
Morphology and structural characterization of MgAl_2_O_4_ and MgAl_2_O_4_:Mn^4+^ nanowire membranes. a) Surface SEM image of MgAl_2_O_4_ nanowire membrane. b) Surface SEM image of MgAl_2_O_4_:Mn^4+^ nanowire membrane. c) Diameter distribution of MgAl_2_O_4_:Mn^4+^ nanowires. d) Cross‐sectional SEM image of MgAl_2_O_4_:Mn^4+^ nanowire membrane. e) XRD pattern of MgAl_2_O_4_ and MgAl_2_O_4_:Mn^4+^ nanowires. f) EDS images of single MgAl_2_O_4_:Mn^4+^ nanowire. g–i) TEM images of single MgAl_2_O_4_:Mn^4+^ nanowire.

**Figure** **2**a shows a scanning electron microscopy (SEM) image of the MgAl_2_O_4_ membrane under high magnification, which clearly shows crosslinked, dense and uniform nanowires with an average diameter of 53.69 nm, ranging from 20 to 90 nm (Figure S9, Supporting Information). Compared to MgAl_2_O_4_ nanowires, the distribution and diameters of MgAl_2_O_4_:Mn^4+^ nanowires are almost unchanged (Figure 2b,c). The cross‐sectional SEM image of the MgAl_2_O_4_:Mn^4+^ nanowire membrane shows that the thickness of the MgAl_2_O_4_:Mn^4+^ nanowire membrane is 50 µm (Figure 2d). The X‐ray powder diffraction (XRD) patterns of MgAl_2_O_4_ and MgAl_2_O_4_:Mn^4+^ are shown in Figure 2e. MgAl_2_O_4_ exhibits thirteen peaks at 19.0°, 31.2°, 36.8°, 38.5°, 44.8°, 55.6°, 59.3°, 65.2°, 68.6°, 74.1°, 77.3°, 78.4°, and 85.7°, corresponding to (1 1 1), (2 2 0), (3 1 1), (2 2 2), (4 0 0), (4 2 2), (5 1 1), (4 4 0), (5 3 1), (6 2 0), (5 3 3), (6 2 2), and (5 5 1), well matching the MgAl_2_O_4_ spinel phase (JCPDS #21‐1152). The diffraction peaks of MgAl_2_O_4_:Mn^4+^ are generally comparable to those of MgAl_2_O_4_ but slightly shift, as shown in Figure S10, Supporting Information. This change can be explained by the Bragg equation (2*d* sin*θ* = *nλ*). Mn^4+^ with a smaller ion radius (0.053 nm) replaces Al^3+^ with a larger ion radius (0.057 nm), causing a decrease in *d* (interplanar spacing) and thus an increase in *θ*. The refined lattice parameters for MgAl_2_O_4_ and MgAl_2_O_4_:Mn^4+^ are 8.08380 and 8.07600 Å, respectively, further indicating Mn^4+^ with a smaller ion radius replaces Al^3+^ with a larger ion radius. Transmission electron microscopy (TEM) analysis further verified the doping of Mn^4+^ and the crystallinity of MgAl_2_O_4_:Mn^4+^. The elemental mapping images (Figure 2f) and content graph (Figure S11, Supporting Information) of a single MgAl_2_O_4_:Mn^4+^ nanowire demonstrate that Mg, Al, O, and Mn were uniformly distributed. This confirms that Mn^4+^ was uniformly doped in MgAl_2_O_4_. Continues string of grains reveals a high crystallinity of the nanowires, which enables good mechanical stability of the FMPE (Figure 2g). The clear lattice fringe with a *d*‐spacing of 0.458 nm measured from the high resolution (HR)‐TEM image (Figure 2h,i) corresponds to the (111) plane of MgAl_2_O_4_:Mn^4+^, which is smaller than the standard interplanar spacing of MgAl_2_O_4_ (0.466 nm), further supporting the XRD results.

The SEM image of MgAl_6_O_10_ nanowires is shown in Figure S12, Supporting Information, and the average diameter of MgAl_6_O_10_ nanowires is 39.78 nm (Figure S13, Supporting Information), thinner than that of MgAl_2_O_4_ and MgAl_2_O_4_:Mn^4+^nanowires due to the lower Mg element content. The XRD pattern of the MgAl_6_O_10_ nanowire membrane is shown in Figure S14, Supporting Information. The peak shifting between MgAl_2_O_4_ and MgAl_2_O_4_:Mn^4+^ is caused by the low content of Mg^2+^ (0.072 nm). The elemental mapping images (Figure S15, Supporting Information) and HR‐TEM image content graph (Figure S16, Supporting Information) of a single MgAl_6_O_10_ nanowire demonstrate that Mg, Al, and O were uniformly distributed and well‐crystallized similar to the MgAl_2_O_4_:Mn^4+^nanowires.

### 2.3 Preparation and Characterization of FMPE

**Figure 3:**
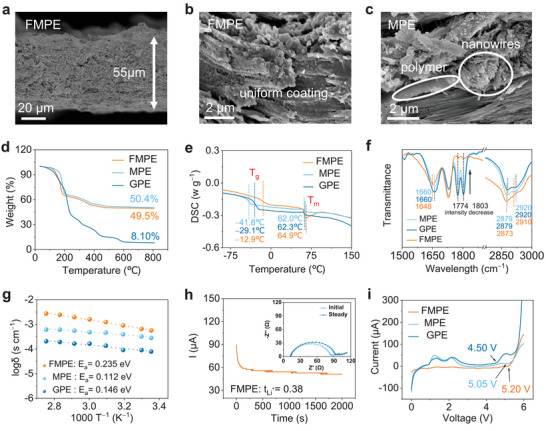
Physicochemical and electrochemical characterization of different membranes. a) Cross‐sectional SEM image of FMPE membrane. Cross‐sectional SEM image of b) FMPE and c) MPE. d) TGA plots of FMPE, MPE, and GPE. e) DSC plots of FMPE, MPE, and GPE. f) FTIR spectra of FMPE, MPE, and GPE. g) Ionic conductivities of FMPE, MPE, and GPE membranes. h) The chronoamperometry profile of Li||Li symmetrical cell using FMPE under a polarization voltage of 10 mV and the EIS before and after the polarization. i) Electrochemical stability window of FMPE, MPE, and GPE determined by LSV.

The FMPE membrane was fabricated by photocuring under 365 nm UV light. The cross‐sectional SEM image of the FMPE membrane displays a thickness of approximately 55 µm (**Figure** **3**a), and PEO is seamlessly coated on nanowires, improving the interfacial compatibility between the ceramic and PEO matrix (Figure 3b). In contrast, in MPE, the PEO blocks fill in between the MgAl_6_O_10_ nanowires clusters and loosely cover their surfaces due to the poor interfacial compatibility between PEO and MgAl_6_O_10_ (Figure 3c). In addtion, the GPE （without inorganic fillers）is very dense (Figure S17, Supporting Information), which indicates the UV‐curing method is a prolonging of the Li^+^ transport distance and poor ion transport. After exposure to 350 °C for 2 h, FMPE maintains the original circular shape owing to the strong interaction between the 3D interconnected nanowire network and PEO, while MPE and GPE sharply shrink due to the decomposition of PEO (Figure S18, Supporting Information). The outstanding thermal stability of FMPE could avoid short circuits in the practical application of LMBs.

Thermogravimetric analysis (TGA) plots of GPE, MPE, and FMPE are shown in Figure 3d. The decomposition of PEO starts at 205 °C, and the carbon residue is approximately 8.10% above 600 °C. Compared to GPE, MPE and FMPE exhibit lower decomposition temperatures (202 and 186 °C), indicating that more amorphous regions exist in the CGPEs. The contents of MgAl_2_O_4_:Mn^4+^ (49.5 wt.%) and MgAl_6_O_10_ (50.4 wt.%) nanowires not only ensure good flame retardancy and high rigidity but also provide numerous ceramic‐polymer interfaces that facilitate PEO gridding. Differential scanning calorimetry (DSC) plots of GPE, FMPE, and MPE are shown in Figure 3e. The glass transition temperature (*T*_g_) of GPE is −29.1 °C, and the melting temperature (*T*_m_) is 62.3 °C. After adding MgAl_6_O_10_ nanowires, *T*_g_ and *T*_m_ decrease to −41.8 and 62.0 °C, respectively, while with the addition of MgAl_2_O_4_:Mn^4+^ nanowires, *T*_g_ and *T*_m_ increase to −12.9 and 64.9 °C, respectively. This implies that the MgAl_6_O_10_ nanowires only act as plasticizers to impede the crystallization of the PEO chain, thus reducing the *T*_g_ and *T*_m_ of the PEO matrix. Nevertheless, the PEO chains wrap so tightly on the MgAl_2_O_4_:Mn^4+^ nanowires that a higher temperature is needed for segmental relaxation. To analyze the interfacial compatibility of the composite electrolytes, Fourier transform infrared (FTIR) spectra were employed (Figure 3f). The peaks at 2879 and 2920 cm^−1^ correspond to the C—H stretching vibration of the PEO. Compared to GPE, the band of C—H in the FMPE shifts from 2879 to 2873 cm^−1^, and 2920 to 2910 cm^−1^, while there is scarcely any peak shift in the MPE (Figure S19a, Supporting Information). The decrease in wavenumber (approximately 10 cm^−1^) of FMPE indicates the strong interaction between MgAl_2_O_4_:Mn^4+^ nanowires and the PEO. The detected peaks at 1660, 1774, and 1803 cm^−1^ assigned to the stretching of C=O belong to PEO monomer. Compared to GPE, the peak in the FMPE shifts from 1660 to 1648 cm^−1^, and the intensity of the peaks at 1774 and 1803 cm^−1^ sharply decreases, while there is no peak shift in the FTIR spectrum of MPE (Figure S19b, Supporting Information), implying the improvement of the interfacial interaction. The ionic conductivity of FMPE at different temperatures was measured via electrochemical impedance spectroscopy (EIS), and the Arrhenius plots are shown in Figure 3g. The conductivity of FMPE is calculated to be 5.66×10^−4^ S cm^−1^, which is higher than that of MPE (2.83×10^−4^ S cm^−1^) and GPE (7.85×10^−5^ S cm^−1^) at 25 °C. Notably, the amounts of LE absorbed in the three kinds of membranes are almost the same (≈ 10 µL, Table S1, Supporting Information), approaching all‐solid‐state electrolytes. Compared to GPE, the activation energy of MPE decreases from 0.146 to 0.112 eV because MgAl_6_O_10_ nanowires hinder PEO crystallization and enable segmental relaxation to increase the Li^+^ hopping probability. In addition, the activation energy of FMPE increases to 0.235 eV because the mobility of the chains is restricted in the tight PEO interphase, reducing the Li^+^ hopping probability. This indicates that although PEO chains wrapped tightly around the MgAl_2_O_4_:Mn^4+^ nanowires need a higher activation energy to transfer Li^+^, the 3D interconnected MgAl_2_O_4_:Mn^4+^ nanowire‐PEO interfaces contribute to the formation of the gridded PEO, thus provides a directional migration pathway for Li^+^ transfer between positive and negative anodes, contributing to the highest ionic conductivity of FMPE. In addition, the Li‐ion transference number (*t*_Li_^+^) of FMPE (0.38) (Figure 3h) is the same as the MPE (0.38) but is higher than that of GPE (0.19) (Figure S20, Supporting Information) because the ceramic nanowires can effectively immobilize PF_6_^−^ and endow Li^+^ in PEO matrix. As shown in Figure 3i, linear sweep voltammetry (LSV) was conducted to confirm the electrochemical window of different GPEs. The oxidation peaks observed between 1.00 and 3.00 V are due to the LE decomposition (Figure S21, Supporting Information). FMPE maintains a widely stable electrochemical window from 3.00 to 5.20 V, while GPE and MPE are only stable from 3.00 to 4.50 V and 3.00 to 5.05 V, respectively. The high stability of FMPE at high voltage enables it to work in high‐voltage batteries.

### 2.4 Li^+^ Conduction Mechanism in FMPE

**Figure 4:**
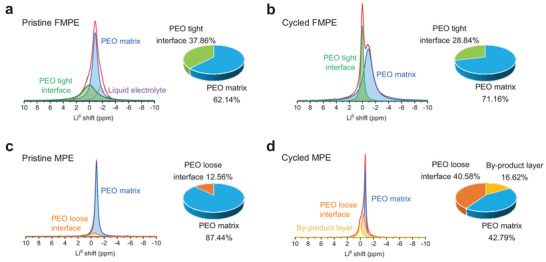
Li^+^ transport behavior within the electrolytes. ^6^Li NMR spectra and the quantified contributions to Li^+^ transport of FMPE a) before and b) after cycling of ^6^Li|FMPE|^6^Li symmetric cell. ^6^Li NMR spectra and the quantified contributions to Li^+^ transport of MPE c) before and d) after cycling of ^6^Li |MPE|^6^Li symmetric cell.

High‐resolution solid‐state Li nuclear magnetic resonance (NMR) technique was performed to give more information about the Li^+^ migration behavior within the composite electrolytes.^[^ ^36^ ^]^ First, ^6^Li NMR spectra were employed to investigate the Li local environment in pristine electrolytes. The Li local environment of PEO matrix was calibrated in the spectrum of GPE, which exists a narrow peak resonance at −0.7 ppm (Figure S22, Supporting Information). Three peaks corresponding to three different Li^+^ environments are identified by fitting the asymmetric peak in the spectrum of FMPE (**Figure** **4**a). The peaks at 0, −0.88, and −1.72 ppm are assigned to the Li^+^ coordinated with the PEO tight interface, the PEO matrix, and the electrolyte solvent,^[^ ^37^ ^]^ respectively. The integral areas of peaks ascribed to PEO tight interface and PEO matrix are 37.86% and 62.14%, respectively. Subsequently, a ^6^Li|FMPE|^6^Li symmetric cell was assembled and charged/discharged for 1 h and 30 times at a current density of 0.2 mA cm^−2^. The Li^+^ transport pathway in the FMPE was determined with ^6^Li →^7^Li tracer‐exchange NMR. After the exchange behavior between ^6^Li and ^7^Li, the integral areas of peaks ascribed to PEO tight interface and PEO matrix is 28.84% and 71.16%, respectively (Figure 4b), which indicates ^6^Li ions preferentially transport through the PEO matrix as the ^6^Li signal is enriched by 9.02%, whereas the ions transport in PEO tight interface decreases from 37.86% to 28.84%.^[^ ^38^ ^]^ The above NMR analysis of FMPE is consistent with the result that Li^+^ conduct in the gridded PEO matrix, thus providing a directional Li^+^ migration pathway and uniform Li^+^ flux between positive and negative anodes, contributing to the highest ionic conductivity of FMPE and the dendrite‐free deposition on the metallic anode.

In the ^6^Li spectrum of pristine MPE (Figure 4c), there exists two peaks at −0.44 and −0.82 ppm, which assigns to the PEO loose interface and the PEO matrix, respectively. It is worth noting that the integral area of the PEO loose interface is only 12.56%, 25.3% less than the integral area of PEO tight interface of pristine FMPE, which is owing to the poor interfacial compatibility and nanowire agglomeration of MPE. As shown in Figure 4d, after the exchange behavior between ^6^Li and ^7^Li, new peak accounts for 16.62% at −0.01 appeared, which can be corresponding to the Li^+^ in the by‐product layer derived from the side reaction between organic groups suspended on the surface of the nanowire and the carbonate solvents in LE. Furthermore, ^6^Li ions preferentially transport through the PEO loose interface as the ^6^Li signal is enriched by 28.02%, whereas the ions transport in PEO matrix decreases from 87.44% to 42.79%. Li^+^ migrates from PEO matrix to PEO loose interface indicating the more diverse Li^+^ conduction pathways within the MPE, and the longer distance Li^+^ transfer between positive and negative anodes, thus resulting in the low ionic conductivity of MPE. Furthermore, the diverse Li^+^ conduction pathways may result in chaotic Li^+^ flux and the formation of Li dendrites.

### 2.5 Electrochemical and Battery Performances

**Figure 5:**
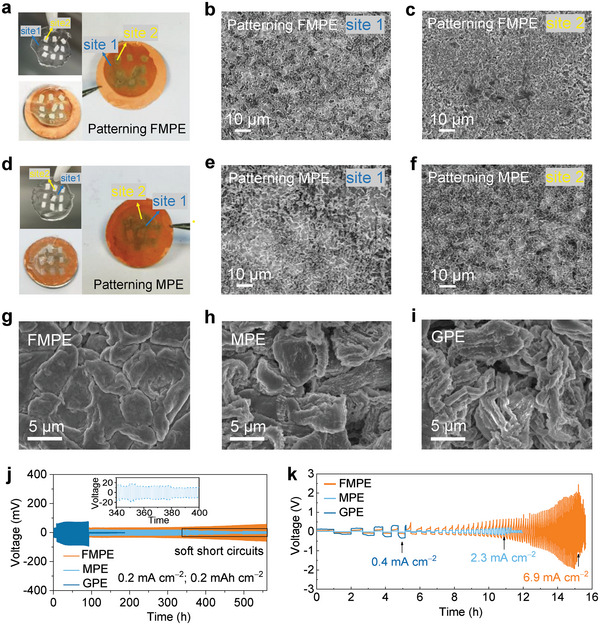
Lithium plating/stripping behavior with different electrolytes. a–c) Surface photographs and SEM images of Li metal morphology deposited on Cu foils of 0.2 mAh cm^−2^ areal capacity using patterning FMPE. d–f) Surface photographs and SEM images of Li metal morphology deposited on Cu foils of 0.2 mAh cm^−2^ areal capacity using patterning MPE. Surface SEM images of Li metal morphology deposited on Cu foils of 3 mAh cm^−2^ areal capacity using g) FMPE, h) MPE, and i) GPE. j) Galvanostatic cycling curves of Li||Li symmetrical cells using FMPE, MPE, and GPE at a current density of 0.2 mA cm^−2^. k) Galvanostatic cycling curves at step‐increased current densities of Li||Li symmetric cells assembled with FMPE, MPE, and GPE.

To understand the Li deposition behavior induced by gridded PEO in FMPE, we assembled Li||Cu cells with patterning FMPE and patterning MPE and charged them at a current density of 0.2 mA cm^−2^ with 0.2 mAh cm^−2^. After the deposition, the photographs showed that Li metal deposited on Cu foil presented patterning. More Li metal accumulates deposition under FMPE than under PEO matrix when using patterning FMPE (**Figure** **5**a). The SEM image of site 1 (under PEO) shows that isolated Li clusters were formed and presented “hot spots” for dendritic growth due to the uneven local Li^+^ concentration and current density distributions caused by the unconfined amorphous regions of the PEO (Figure 5b). At site 2 (under FMPE), compact and horizontal Li was formed due to numerous and uniform nucleation sites (Figure 5c). Specifically, the PEO mesh units induce the directional transport of Li^+^ to the copper surface and nucleation in a limited region (Figure S23a, Supporting Information). The photographs of patterning MPE show that Li metal deposited on Cu foil is disorganized (Figure 5d). In terms of the deposited state 1 under the PEO, irregular whisker‐like Li dendrites were loosely isolated on the Cu substrate due to the chaotic Li^+^ flux induced by amorphous PEO (Figure 5e). Similarly, at site 2 under MPE, the uneven Li clusters were widely dispersed due to the heterogeneous Li^+^ distribution (Figure 5f), which derived from the obstacles of inert nanowires clusters, chaotic Li^+^ flux in unconfined amorphous PEO matrix and pores between loose PEO and MgAl_6_O_10_ nanowires (Figure S23b, Supporting Information). When increasing the Li plating capacity to practically necessary 3 mAh cm^−2^, densely plated Li metal was formed using FMPE mainly due to the uniform Li^+^ distribution generated in the confined PEO mesh units, which induced homogeneous deposition of Li on the electrode (Figure 5g). Although the rigid MgAl_6_O_10_ nanowire network in MPE also mechanically blocked Li dendrites to a certain extent, the loose and chaotic Li^+^ channels inside MPE and at the interface of MPE and Cu led to the generation of Li tips, which continued to form Li dendrites (Figure 5h). In contrast, sharp Li dendrites emerged in the Li||Cu cell using GPE because of uneven Li deposition and severe side reactions (Figure 5i). Furthermore, Li symmetric cells were applied for the galvanostatic cycling test at a current density of 0.2 mA cm^−2^ and a capacity of 0.2 mAh cm^−2^. As shown in Figure 5j, the cell with FMPE shows excellent long‐term cycling stability for over 560 h without a short circuit, while the comparative cells with MPE and GPE present a short circuit after 345 and 90 h owing to the growth of Li dendrites, which further suggests that the constructed homogeneous, confined and continuous ion transport channels in gridded PEO can inhibit the side reactions and the growth of Li dendrites. Critical current density (CCD) tests were conducted to obtain the maximum current density under an areal capacity of 0.2 mAh cm^−2^. As shown in Figure 5k and Figure S24, Supporting Information, the CCD (6.9 mA cm^−2^) of the cell using FMPE is 3 and 17 times higher than that of the cells using MPE and GPE, respectively, indicating that improved Li^+^ confinement modulation may induce homogeneous uniform Li deposition and suppress Li dendrite growth to withstand battery short circuit.

**Figure 6:**
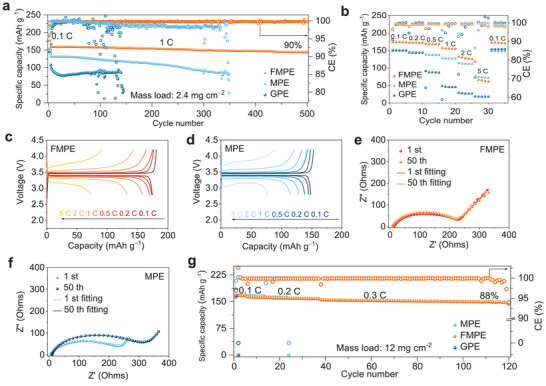
Electrochemical performances of Li||LFP cells with different electrolytes. a) Long‐term cycling performance of Li||LFP cells using FMPE, MPE, and GPE at 1 C under 30 °C. b) Rate performances of Li||LFP cells using FMPE, MPE, and GPE. Mass loading of LFP is 2.4 mg cm^−2^. Charge/discharge curves of Li||LFP cells with c) FMPE and d) MPE from 0.1 to 5 C. Mass loading of LFP is 2.4 mg cm^−2^. EIS plots of Li||LFP cells with e) FMPE and f) MPE after different cycles. g) Long‐term cycling performances of Li||LFP cells using FMPE, MPE, and GPE from 0.1 to 0.3 C under 30 °C. Mass loading of LFP is 12 mg cm^−2^.

To further evaluate the electrochemical performance of different GPEs in full cells, Li|FMPE|LFP, Li|MPE|LFP, and Li|GPE|LFP cells were assembled (the mass loading of LFP was 2.4 and 12 mg cm^−2^) and tested with a voltage range from 2.8 to 4.2 V. As shown in **Figure** **6**a, the Li|FMPE|LFP cell manifests extremely stable and brilliant discharge capacity retention (from 159.8 to 144.1 mAh g^−1^, 90%) even after 500 cycles, in stark contrast to the Li||LFP full cell using MPE, which only discharges for 350 cycles (from 154.5 to 82.20 mAh g^−1^, 53%), and that using the GPE, which discharges for 142 cycles (from 107.6 to 32.00 mAh g^−1^, 30%). This result highly corresponds to that of Li||Li symmetric cells and further demonstrates that the existence of gridded PEO can significantly improve the long‐term cycling performance of Li||LFP full cells. Figure 6b compares the rate performances of Li||LFP cells using FMPE, MPE, and GPE. The specific capacities of the battery using FMPE at 0.1 and 0.2 C are 174.2 and 172.9 mAh g^−1^, respectively, outclassing those of its counterparts. Particularly, as the current density increases to 2 C, the discharge capacity of the Li|FMPE|LFP cell is higher than that of the Li|MPE|LFP cell and almost 4.7 times higher than that of the Li|GPE|LFP cell. When the rate further increases to 5 C, the discharge capacity of Li|FMPE|LFP is almost the same as that of Li|MPE|LFP and still higher than that of the Li|GPE|LFP cell. Moreover, the reversible capacity of the Li|FMPE|LFP cell reaches 172.9 mAh g^−1^ when the current density returns to 0.1 C. Furthermore, the (dis)charge overpotentials of the Li|FMPE|LFP cell at different C‐rates are much lower than those of Li|MPE|LFP and Li|GPE|LFP (Figure 6c,d and Figure S25, Supporting Information). The EIS and simulated results of the Li||LFP full cell with FMPE, MPE, and GPE after different numbers of cycles are shown in Figure 6e,f, Figure S26, Figure S27 and Table S2, Supporting Information. Both the charge transfer resistance (*R*_ct_) and solid electrolyte resistance (*R*_SEI_) of the Li|FMPE|LFP and Li|MPE|LFP cells are smaller than that of Li|GPE|LFP after different numbers of cycles due to the introduction of nanowires into GPE. Specifically, comparing the first cycle with the 50th cycle, both the *R*_ct_ and *R*_SEI_ of the Li|FMPE|LFP cell are slightly decreased (*R*_ct_ from 76.86 to 72.08 Ω, *R*_SEI_ from 132.5 to 122.8 Ω), while those of the Li|MPE|LFP cell are dramatically increased (*R*_ct_ from 167.1 to 198.4 Ω, *R*_SEI_ from 59.84 to 116.4 Ω), indicating that the solid electrolyte interphase (SEI) formed on the Li metal using FMPE is more stable than that formed using MPE. In addition, as the LFP loading increases from 2.4 to 12 mg cm^−2^, the discharge capacity of the Li|FMPE|LFP cell is 162.6 mAh g^−1^ with a capacity retention of 88% after 120 cycles at different C‐rates, which is outstanding than that of Li|GPE|LFP only cycling for 1 cycle and the Li|MPE|LFP cell cycling for 25 cycles (Figure 6g). The 12 mg cm^−2^ LFP cathode required a small amount of LE to infiltrate the cathode and reduce the interfacial impedance. Such a significant improvement in the cycling performance and low polarization indicate that the gridded PEO in FMPE contributes to the uniform distribution of Li^+^, which improves the interfacial compatibility with the Li metal anode and forms an extremely steady SEI film to suppress Li dendrites. The electrochemical performances of Li|FMPE|LFP cells are much better than those of other recently reported solid‐state batteries (Table S3 and Figure S28, Supporting Information). To illustrate the high stability of FMPE when working in high‐voltage batteries. Li|FMPE|LiNi_0.8_Co_0.1_Mn_0.1_O_2_ (NCM811) and Li|MPE|NCM811 were assembled and tested with a voltage range from 2.8 to 4.5 V. As shown in Figure S29, Supporting Information, the cell with FMPE presents higher capacity retention than that with MPE, indicating that FMPE has better compatibility with NCM811. The voltage profiles of Li||NCM811 cells have been added in Figure S30, Supporting Information. The Li|FMPE|NCM811 exhibits lower polarization and higher specific capacity. In addition, we also compared the XRD patterns of the NCM811 cathode after cycling with FMPE and MPE (Figure S31, Supporting Information). The intensity ratio between diffraction peaks corresponding to the lattice planes (003) and (104) (I_(003)_/I_(104)_) can be used to reflect the degree of Li/Ni mixing.^[^ ^39^ ^]^ Compared with NCM811 circulating with MPE, the I_(003)_/I_(104)_ ratio in NCM811 circulating with FMPE is closer to the fresh NCM811, so it can be concluded that the FMPE is beneficial to maintain the highly ordered hexagonal layered structure of the NCM811 cathode material and reduce the degree of Li/Ni mixing during cycling. Moreover, XPS analysis was carried out to characterize the NCM811 cathode after cycles with FMPE and MPE (Figure S32, Supporting Information). Compared to the NCM811 cathode using MPE, less Ni^2+^ was exhibited in the Li|FMPE|NCM811 cell after etching for 60 s, demonstrating less formation of the irreversible NiO‐like rock‐salt structure on the cathode surface,^[^ ^40^ ^]^ further indicating that FMPE has better compatibility with NCM811.

**Figure 7:**
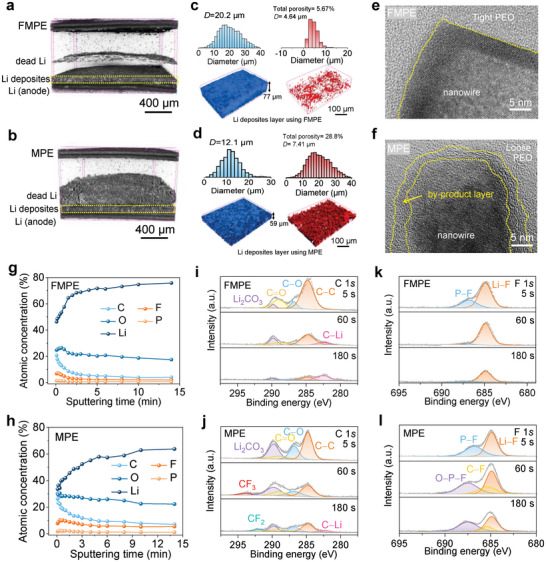
Physicochemical characterization of cycled Li anode and electrolytes in Li||LFP cells. Regional tomography slice of the cell at charge state after 25 cycles using a) FMPE and b) MPE. 3D maps and diameter distribution of the particles and pores in Li deposit layer using c) FMPE and d) MPE, where the particles are shown in blue, and the pores are shown in red. TEM images of the e) FMPE and f) MPE membranes after 5 cycles. Atomic concentration varies with sputtering time of Li anodes after 200 cycles using g) FMPE and h) MPE. C1*s* and F1*s* XPS spectra of Li anodes using i,k) FMPE and j,l) MPE.

Micro‐X‐ray computed tomography (Micro‐CT) was carried out to study the Li plating process after 25 cycles in Li|MPE|LFP, and Li|FMPE|LFP cells (Mass loading of LFP is 12 mg cm^−2^). The tomographic measurements record transmission images at different viewing angles and reconstruct them into a 3D representation of the sample shown in **Figure** **7**a,b. In the reconstructed 3D tomograms, the boundaries among the electrolyte membrane, dead Li layer, Li deposits layer, and the Li anode layer were observed. The porous structure and particle size of the dead Li layer and Li deposits layer can be visualized in 3D. The thickness of the dead Li layer in Li|MPE|LFP cell is 200 µm, four times thicker than using FMPE (Figure S33, Supporting Information). Such a thick dead Li layer in Li|MPE|LFP cells indicating that many Li dendrites formed and electrically isolated, corresponds to the capacity fading and lifespan declining of Li|MPE|LFP cells. 3D maps and diameter distribution of the particles and pores in the Li deposit layer using FMPE and MPE are shown in Figure 7c,d. The total porosity of the Li deposit layer in Li|FMPE|LFP cell is 5.67%, almost six times less than that of using MPF (28.8%). Correspondingly, the average diameter of the particle size is 20.2 µm when using FMPE, almost two times larger than the counterpart (12.1 µm). Such a dense and uniform Li deposit layer in Li|FMPE|LFP cell corresponds to the densely plated Li metal, indicating that the gridded PEO providing directional and homogeneous Li^+^ flux can induce dendrite‐free Li deposition to receive stable recycling ability.

To further understand the effect of nanowire‐PEO interfaces on battery performance, FMPE and MPE membranes in the Li||LFP full cell after 5 cycles were characterized with TEM (Figure 7e,f). In the FMPE membrane, there is a clear boundary between the inorganic nanowires and tight PEO due to the tight nanowire‐PEO interfaces preventing the LE from decomposing, which corresponds with the electrochemical performance of the Li||LFP full cell with a long lifespan. However, in the MPE membrane, a by‐product layer can be observed between inorganic nanowires and loose PEO due to the loose nanowire‐PEO interfaces that exist pores that are rich in LE and thus arising side reaction between organic groups suspended on the surface of the nanowire and the carbonate solvents in LE. Such a reaction seriously consumes the LE and deteriorates the performance of Li|MPE|LFP cell.

X‐ray photoelectron spectroscopy (XPS) analysis with depth profiling was carried out to characterize the chemical composition of the SEI formed on Li metal anodes after 200 cycles in Li|MPE|LFP and Li|FMPE|LFP cells. The atomic concentration results show that the content of C when using MPE is always higher than that when using FMPE (Figure 7g,h), indicating less solvent molecule decomposition and a more stable SEI in the Li|FMPE|LFP cell. The C 1*s* spectra of Li anodes using FMPE and MPE shown in Figure 7i,j were fitted to C—C (284.8 eV), C—O (286.8 eV), C=O (289.0 eV), Li_2_CO_3_ (289.9 eV), Li—C (282.5 eV), CF_3_ (294.0 eV), and CF_2_ (292.0 eV).^[^ ^41^, ^42^, ^43^ ^]^ Compared to the SEI film using FMPE, extra peaks, such as those of CF_3_ and CF_2_, are exhibited in the Li|MPE|LFP cell when etching to 60 and 180 s, respectively, demonstrating more decomposition of organic solvent in the cell using MPE. Notably, the abundance of Li_2_CO_3_ when using MPE is expected to result in easy rupture of the SEI due to the brittle nature of Li_2_CO_3_ and lack of adhesion with Li metal.^[^ ^42^ ^]^ In contrast, the Li_2_CO_3_ content when using FMPE is always less throughout the increasing etching time, further indicating the stability of the SEI. Furthermore, with increasing etching time, the C—Li peak appears after 60 s when using FMPE, while it appears after 180 s for the counterpart, indicating that the SEI film in the Li|FMPE|LFP cell is much thinner and denser. In the F 1*s* spectra of the SEI layers (Figure 7k,l), the peaks at 684.9, 685.4, 686.8, and 687.5 eV are attributed to Li—F, C—F, P—F, and O—P—F, respectively.^[^ ^44^, ^45^, ^46^ ^]^ Compared to the SEI film using FMPE, extra peaks, such as those of C—F and O—P—F, are exhibited in the Li|MPE|LFP cell when etching to 60 s, and the decline in the intensity of these two peaks is not obvious until etching for 180 s, corresponding to more decomposition of LiPF_6_ and organic solvent. Notably, the intensity of the Li—F peak for FMPE decreases 57% when etching from 5 to 180 s, while that for the counterpart only decreases 40%, further indicating that the SEI in the Li|FMPE|LFP cell is much thinner and more stable. Therefore, FMPE provides directional Li^+^ pathways generated in the gridded PEO to induce homogeneous deposition of Li on the electrode and effectively regulate the composition and structure of SEI films, thus successfully suppressing Li penetration.

## 3 Conclusion

In this work, we use a luminescent ceramic filler (MgAl_2_O_4_:Mn^4+^ nanowires) as an internal light source to reduce the decomposition energy barrier of photoinitiators (BAPO) and contribute to preferential nucleate and growth of polymer monomers, leading to a dense PEO layer wrapping around the nanowires and inducing a gridded PEO with directional and uniform Li^+^ conduction pathways. The innovative design results in a high ionic conductivity of 5.66×10^−4^ S cm^−1^ at 25 °C and high voltage stability up to 5.20 V, and excellent cycling performance (90% capacity retention after 500 cycles at 1 C) is achieved for the Li||LFP full cell with FMPE. Furthermore, a full cell with a high‐load LFP cathode (12 mg cm^−2^) also demonstrates a high capacity. This work provides a general strategy to overcome the persistent problem of poor interfacial compatibility in composite electrolytes and promote the development of high‐performance solid‐state LMBs.

## 4 Experimental Section

### Materials

Aluminum powder (99.5%), Mg (CH_3_COO)_2_ (99%), and Mn (CH_3_COO)_2_ (99%) were purchased from Aladdin. Li powder was purchased from China Energy Lithium Co., Ltd. Ethanol (>99.8%, HPLC), chlorin e6 (90%), and BAPO (Photoinitiator) (98%) were supplied by Macklin. PEGDA (Mv≈700) was purchased from Sigma. Liquid electrolyte (1 mol LiPF_6_ in ethylene carbonate (EC), dimethyl carbonate (DMC), and ethyl methyl carbonate (EMC), volume ratio 1:1:1 (H_2_O < 10 ppm)) came from Suzhou Qianmin Chemical Reagent Company. The commercial LFP electrode (cathode loading was 12 mg cm^−2^, active material ratio was 96 wt.%, the thickness was 68 µm) was purchased from HF‐Kejing Co., Ltd. The commercial NCM811 and LFP powder were provided by Guangdong Canrd New Energy Technology Co., Ltd. NCM811 cathode with mass loading of 2.0 mg cm^−2^ was prepared by dissolving the mixture 80 wt.% NCM811 powder, 10 wt.% acetylene black, and 10 wt.% polyvinylidene difluoride (PVDF) in N‐methyl‐2‐pyrrolidone (NMP) to form a slurry. LFP cathode with mass loading of 2.4 mg cm^−2^ was prepared by dissolving the mixture 70 wt.% LFP powder, 20 wt.% acetylene black, and 10 wt.% PVDF in NMP to form a slurry. The electrode slurry was then pasted on Al foil (99.35%, 16 µm, HF‐Kejing Co., Ltd., China) and dried at 80 °C under vacuum for 12 h. All materials were used as received.

### Preparation of FMPE, MPE, and GPE

PEGDA (with 1 wt.% photoinitiator BAPO and 0.01 wt.% photosensitizer Ce6) and liquid electrolyte were first mixed in a ratio of 3:10 with stirring overnight to make a homogeneous mixture solution. PEO was coated on MgAl_2_O_4_:Mn^4+^ nanowire membranes by dipping in the mixture solution and followed by photocuring of the polymer with 365 nm UV light (5.5 mW cm^−2^) for 5 min. Using the same method, MPE was obtained. 50 µL precursor solution was taken on a 2 cm teflon mold and photocured to obtain the GPE. Before photocuring, all the processes were conducted in the dark to avoid the influence of natural light. Membranes were cut into pellets with a diameter of 16 mm and then soaked in the liquid electrolyte for 5 min to prepare the GPE, MPE, and FMPE.

### Photolysis Study of BAPO

Photolysis kinetics of BAPO upon 365 nm light and 650 nm light (5 W cm^−2^) irradiation were studied through time‐dependent UV–vis measurements. The BAPO solution, containing 0.4 mg BAPO in 10 mL ethanol solution, was stirred for the photolysis study. Then, the prepared solution was exposed to different irradiation. UV–vis spectroscopies were taken at intervals to observe the photolysis of BAPO. Concentration of the BAPO was calculated according to the Beer–Lambert Law, then ln(*c*) was plotted against *t* to obtain a straight line with a slope of −*k*, and the *k* is the photolysis rate constant.

### Cell Assembly

The LFP cathode (2.4 and 12 mg cm^−2^, diameter of 10 mm) and NCM811 cathode (2.0 mg cm^−2^, diameter of 10 mm) matching commercial Li metal anode (99.95%, 400 µm, China Energy Lithium Co., Ltd.) were assembled into 2032 coin cells in an Ar‐filled glove box (Mikrouna) (H_2_O < 0.1 ppm, O_2_ < 0.1 ppm). Before assembling the full cell, 10 µL of precursor solution was injected onto the LFP electrode (2.4 mg cm^−2^), followed by UV irradiation for 5 min as a preliminary treatment. The Li||LFP full cell with 12 mg cm^−2^ LFP cathode and Li||NCM811 full cell without the above photocuring process. The Li||Li and Li||Cu (9 µm Cu metal foil with a purity of 99.8% came from Guangdong Canrd New Energy Technology Co., Ltd.) coin cells were assembled into 2032 coin cells in an Ar‐filled glove box.

### Material Characterization

SEM images were taken by a Regulus 8230, Hitachi. The crystallographic structures of the samples were examined using XRD (D‐MAX 2200 VPP, RIGAKU) and TEM (Tecnai G2 F30, FEI). The cross‐sections of membranes characterized by AFM were cut by a Leica UC6/FC6 in liquid nitrogen. AFM experiments were performed under ambient conditions using a Dimension Fastscan, Bruker. Micro‐CT experiments with 800 nm spatial resolution were conducted with skyscan2211, Bruker. During the implementation, FMPE and MPE membranes with lithium anodes were packaged with Kapton tape in situ for 3D imaging using X‐ray tomography. XPS measurements were collected on an ESCALab250, Thermo Fisher. The solid‐state NMR was performed in a 14.1 T magnetic field with a Bruker 600 MHz AVANCE III spectrometer. The solid‐state ^6^Li magic angle spinning (MAS) NMR spectra were acquired using a single pulse under the spinning frequency of 25 kHz.

The TGA measurement was conducted with a TG209F1 Libra, Netzsch, from room temperature to 800 °C, at a scanning rate of 10 °C min^−1^. *T*_g_ and *T*_m_ were measured using DSC with a DSC‐204 F, Netzsch, at a scanning rate of 10 °C min^−1^ from −100 to 150 °C. FTIR spectra were attained by a Vertex70 Hyperion3000, Bruker, in the frequency range of 400–4000 cm^−1^. Steady‐state photoluminescence spectra were measured by a fluorescence spectrophotometer (FLS1000, Edinburgh). The UV and visible (UV–vis) spectrum was measured by a Lambda 950, Perkin Elmer. The electrolyte uptake (*η*) was calculated by using Equation (5).

(5) $$\eta \;=\frac{W_{t}-W_{0}}{W_{0}}$$

*W*_0_ and *W*_t_ denote the weights of the membranes before and after soaking in liquid electrolyte for 2 h, respectively.

### Electrochemical Characterization

For ionic conductivity measurements, FMPE, MPE, and GPE were sandwiched by two blocking stainless steel electrodes and then evaluated by EIS over the frequency range of 0.01–250 kHz with a 5 mV AC oscillation (Vertex.One.EIS, Ivium) in the temperature range of 25–90 °C. The ionic conductivity (*σ*) was calculated by using Equation (6).^[^ ^47^ ^]^

(6) $$\sigma \;=\frac{L}{RS}$$

in which *S* is the area of stainless steel, *L* is the thickness of the electrolyte, and *R* is the bulk resistance. The classical Arrhenius relationship was suitable for describing the temperature dependence of *σ* for electrolytes, and the activation energy (*E*_a_) of electrolytes can be calculated from Equation (7).^[^ ^48^ ^]^

(7) $$\sigma \;\left(T\right)=A\;\exp\;\left(-\frac{E_{a}}{RT}\right)$$

in which *T* is the absolute temperature, *E*_a_ is the activation energy, and *A* is a pre‐exponential factor. The lithium‐ion transference number (*t*_Li_^+^) measurement was conducted on Li||Li symmetric cells by using the combined AC impedance and DC polarization. *t*_Li_^+^ can be calculated by using Equation (8).^[^ ^49^ ^]^

(8) $$t_{{\mathrm{Li}}^{+}}=\frac{I_{\mathrm{SS}}\left(\Delta V-I_{0}R_{0}\right)}{I_{0}\left(\Delta V-I_{\mathrm{SS}}R_{\mathrm{SS}}\right)}$$

where *I*_0_ and *I*_S_ represent the currents in the initial and steady states, respectively; *R*_0_ and *R*_S_ are the charge transfer resistances of cells before and after DC polarization, respectively and Δ*V* is the applied voltage (10 mV). LSV was performed using a Li||SS cell from 0 to 6 V at a scanning rate of 1 mV s^−1^. The long‐term cycling and C‐rate tests of Li||Li symmetric cells, Li||LFP full cells, and Li||NCM811 were performed on a battery test system (CT2001A, LAND) at 30 °C. The EIS spectra of Li||LFP cells were measured in a frequency range of 0.01–250 kHz with an AC perturbation of 5 mV (Vertex.One. EIS, Ivium).

## Conflict of Interest

The authors declare no conflict of interest.

## Supporting information

Supporting Information

## Data Availability

The data that support the findings of this study are available from the corresponding author upon reasonable request.
