# Supplementary material for: Directional Ion Transport Enabled by Self‐Luminous Framework for High‐Performance Quasi‐Solid‐State Lithium Metal Batteries
Source: Adv Sci (Weinh). 2022 Dec 11;10(4):2205108. doi: 10.1002/advs.202205108 (PMC9896055; doi:10.1002/advs.202205108)
Supplement: Supplementary file 1 — Supporting Information [file ADVS-10-2205108-s001.pdf]

ADVANCED SCIENCE
Open Access

Supporting Information

for *Adv. Sci.*, DOI 10.1002/advs.202205108

Directional Ion Transport Enabled by Self-Luminous Framework for High-Performance Quasi-Solid-State Lithium Metal Batteries

Siyang Ye, Fei Tian, Kaiyuan Shi, Danni Lei and Chengxin Wang**

Supporting Information

Directional Ion Transport Enabled by Self-luminous Framework for High-performance Quasi-solid-state Lithium Metal Batteries

Siyang Ye, Fei Tian, Kaiyuan Shi, Danni Lei, Chengxin Wang**

State Key Laboratory of Optoelectronic Materials and Technologies, School of Materials Science and Engineering, Sun Yat-sen (Zhongshan) University, Guangzhou 510275, China.

*Corresponding author

E-mail: leidanni@mail.sysu.edu.cn; wchengx@mail.sysu.edu.cn

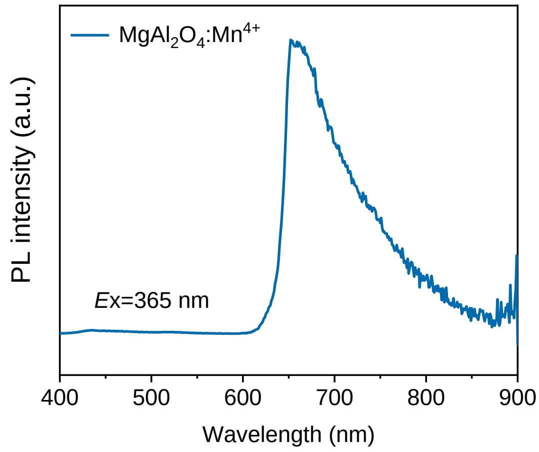

Figure S1. The emission spectrum of $MgAl_2O_4:Mn^{4+}$ measured under a 365 nm UV excitation.

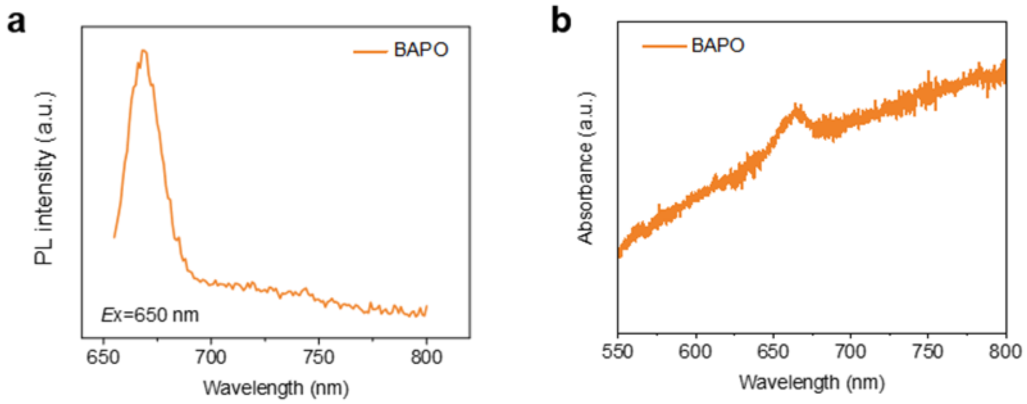

Figure S2. The emission spectrum of BAPO measured under a 650 nm excitation (a). The ultraviolet and visible (UV) spectrum of BAPO/EtOH solution (b).

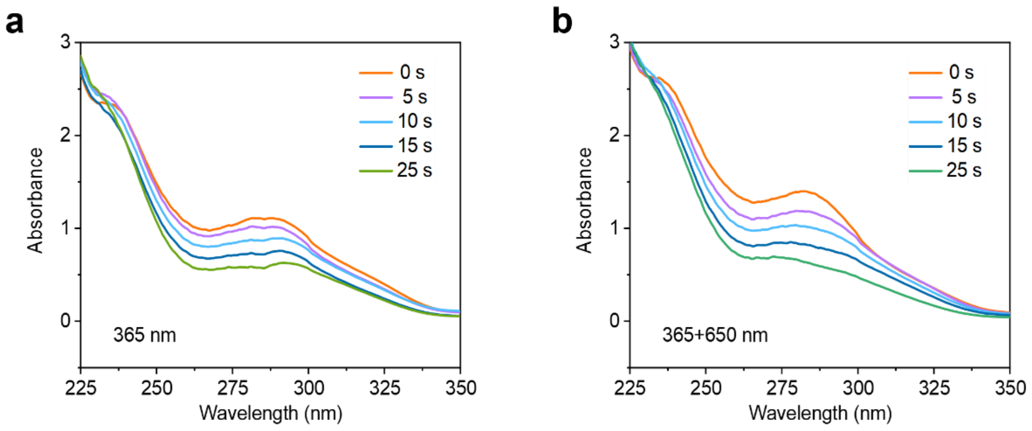

Figure S3. UV−vis absorption evaluation of BAPO real time photodecomposition in ethanol upon irradiation with 365 nm light (a) and irradiation with 365 nm light and 650 nm light (b).

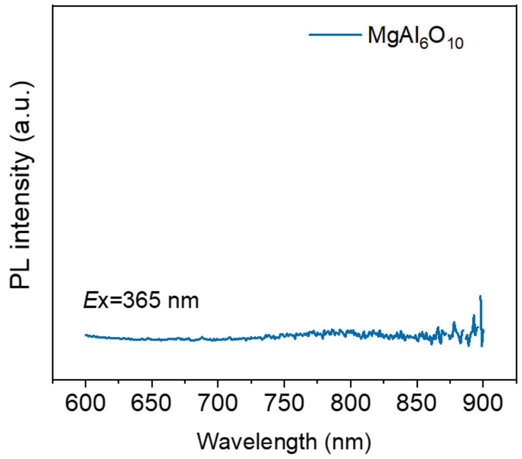

Figure S4. The emission spectrum of $MgAl_6O_{10}$ measured under a 365 nm UV excitation.

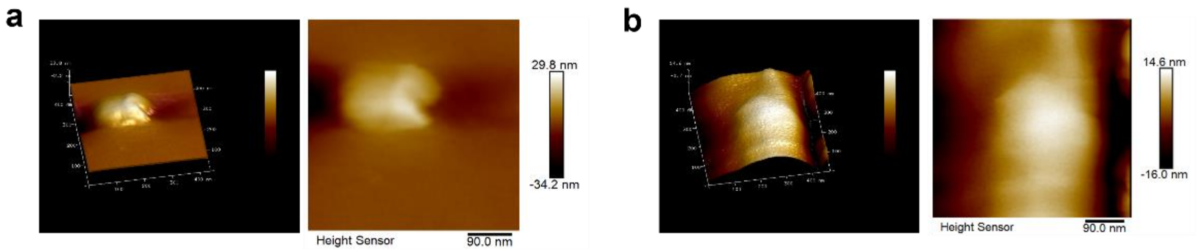

Figure S5. Cross-sectional AFM height images of FMPE (a) and MPE (b).

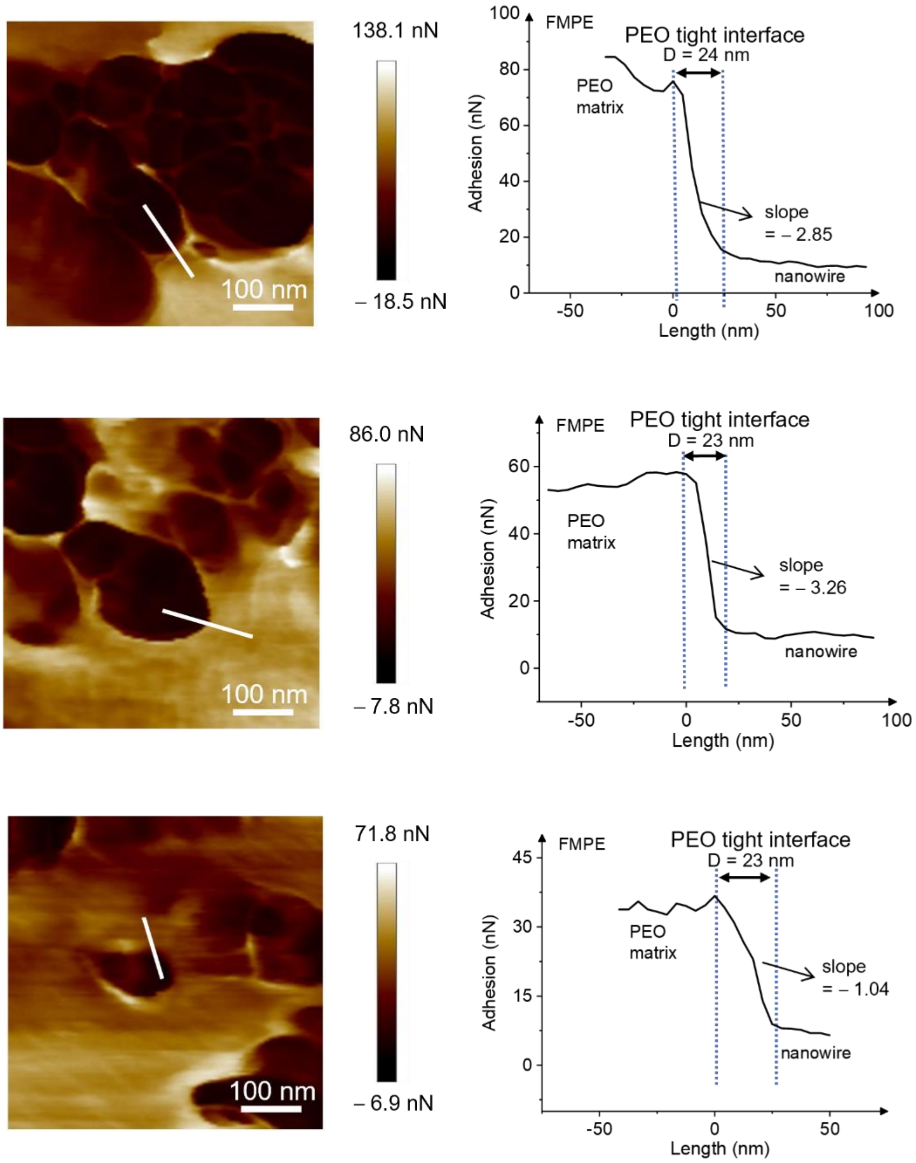

Figure S6. Cross-sectional AFM adhesion image and adhesion distribution curves of FMPE.

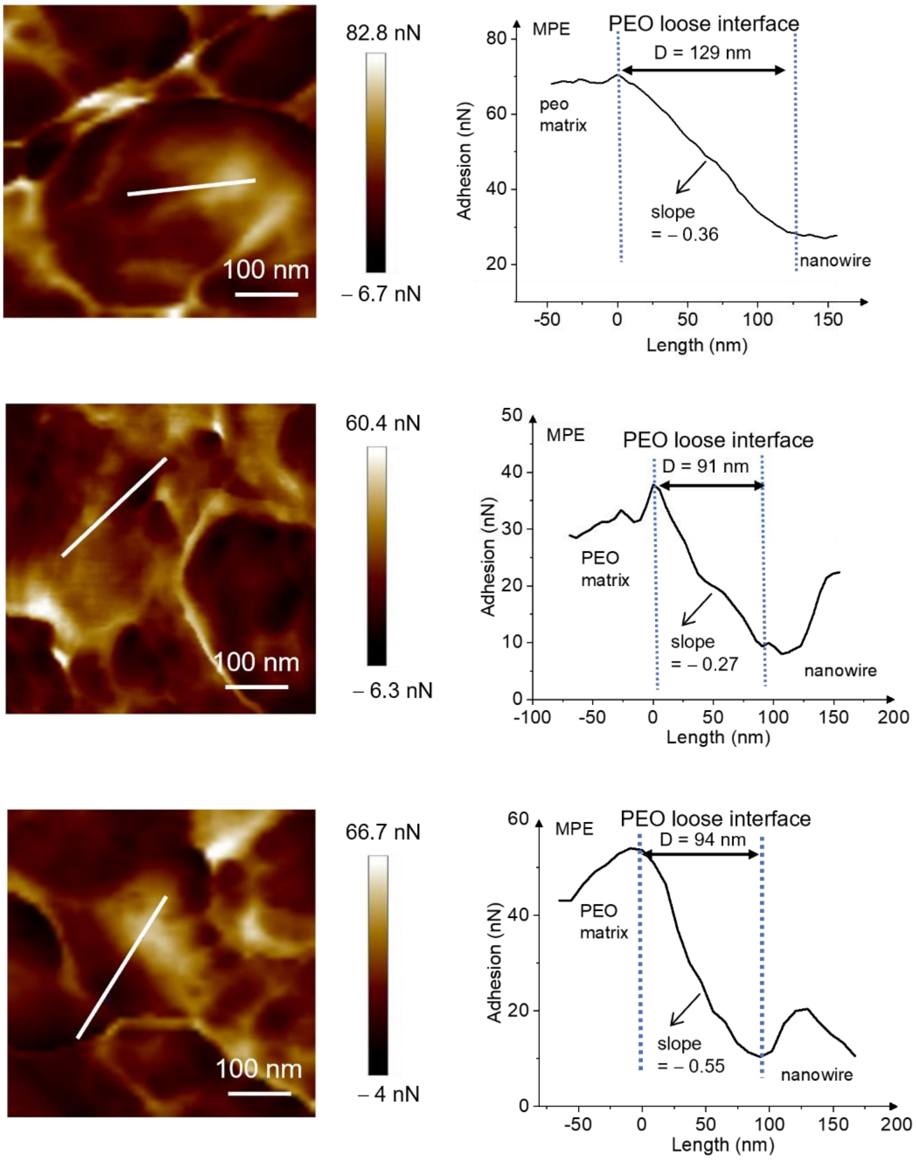

Figure S7. Cross-sectional AFM adhesion image and adhesion distribution curves of MPE.

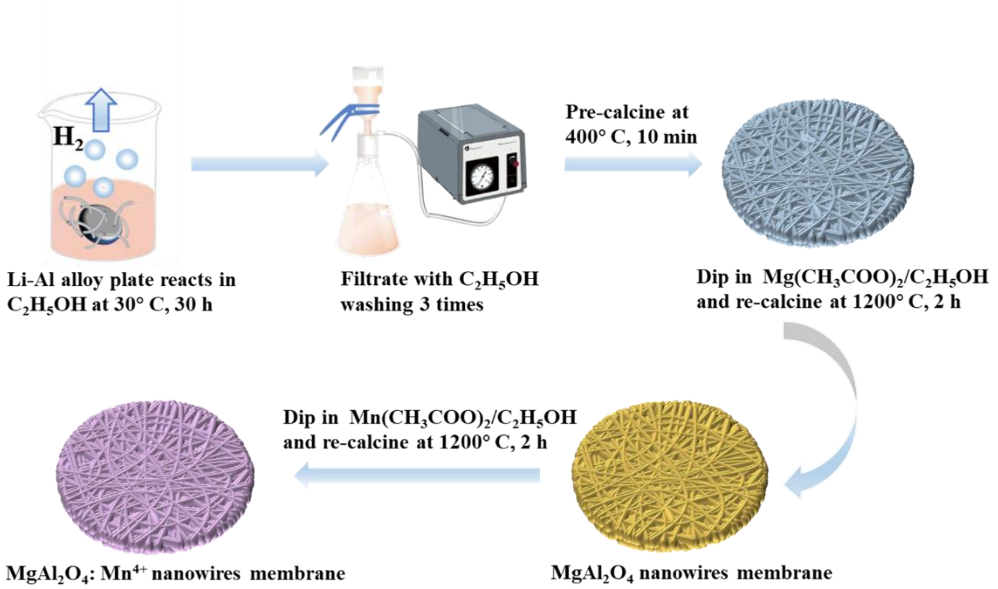

Figure S8. Schematic diagram of preparation process of $MgAl_2O_4$:Mn^{4+} nanowires membrane.

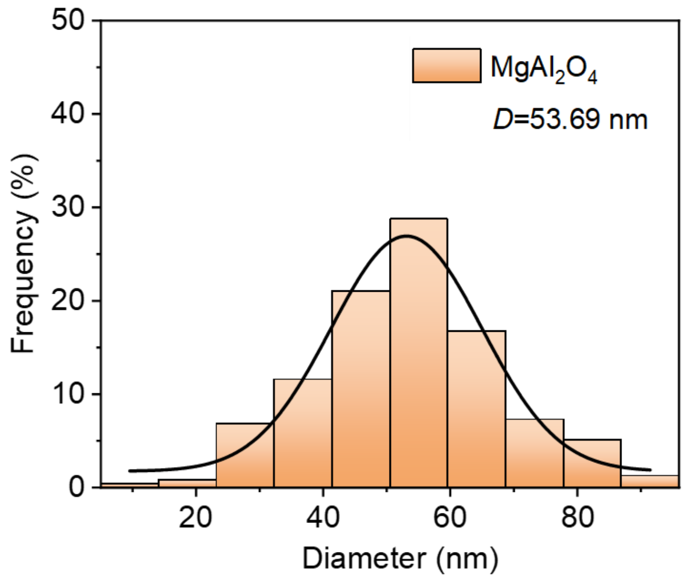

Figure S9. Diameter distribution of $MgAl_2O_4$ nanowires.

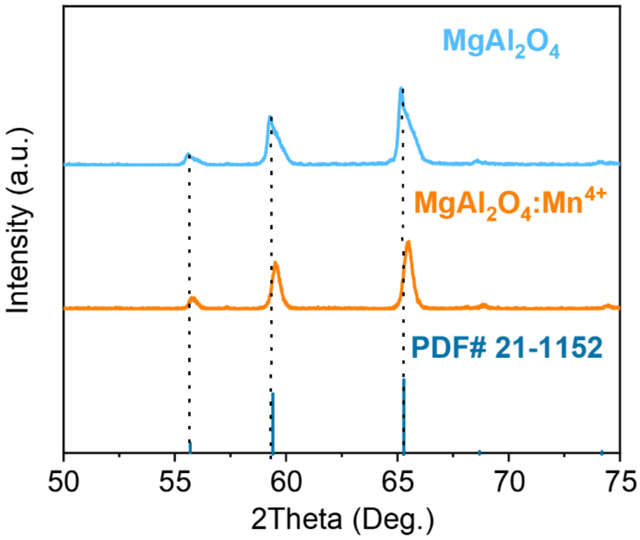

Figure S10. XRD pattern of $MgAl_2O_4$ and $MgAl_2O_4:Mn^{4+}$ nanowires.

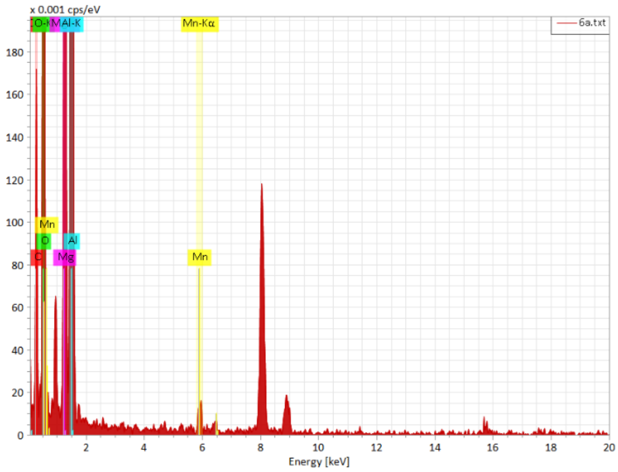

Figure S11. Element content of $MgAl_2O_4:Mn^{4+}$ nanowires.

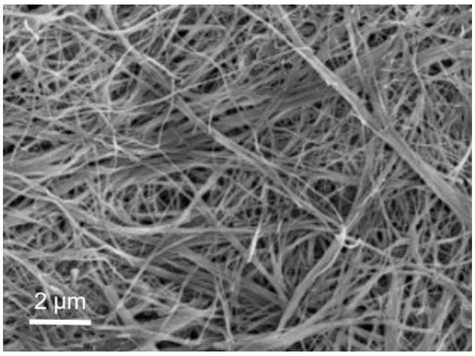

Figure S12. Surface SEM image of $MgAl_6O_{10}$ nanowires membrane.

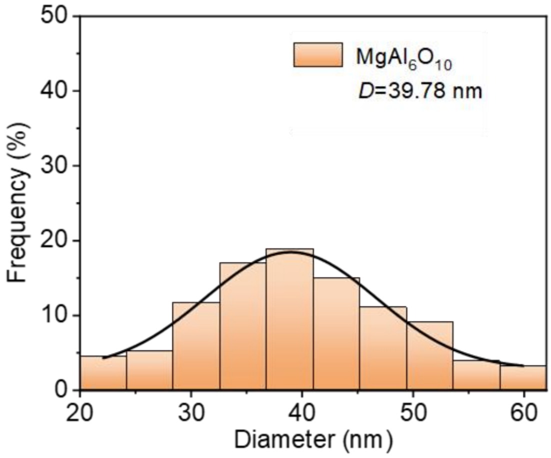

Figure S13. Diameter distribution of $MgAl_6O_{10}$ nanowires.

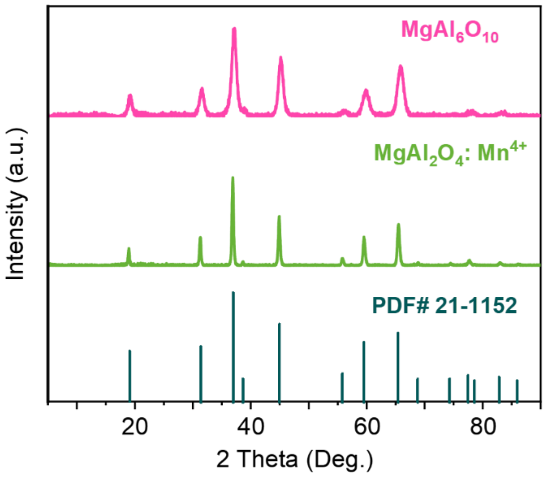

Figure S14. XRD pattern of $MgAl_2O_4:Mn^{4+}$ and $MgAl_6O_{10}$ nanowires.

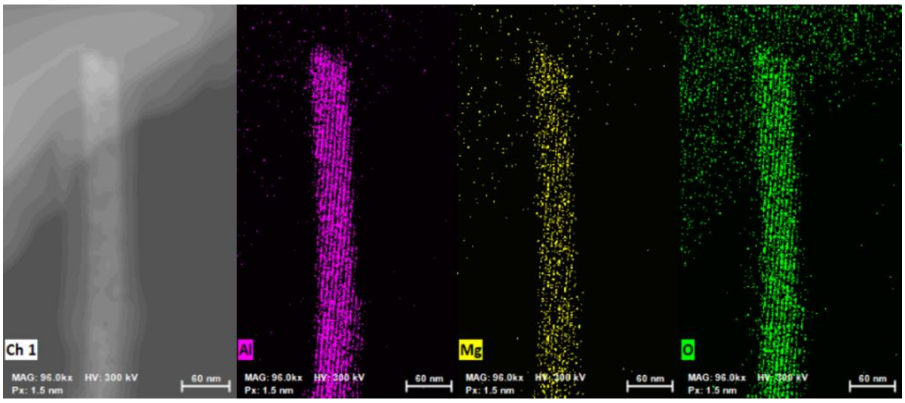

Figure S15. EDS images of single $MgAl_6O_{10}$ nanowire.

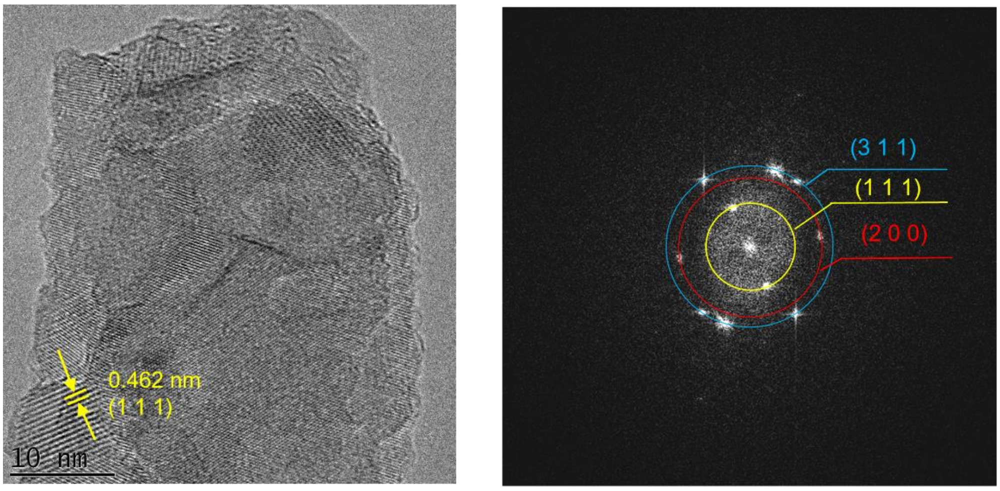

Figure S16. TEM images of single $MgAl_6O_{10}$.

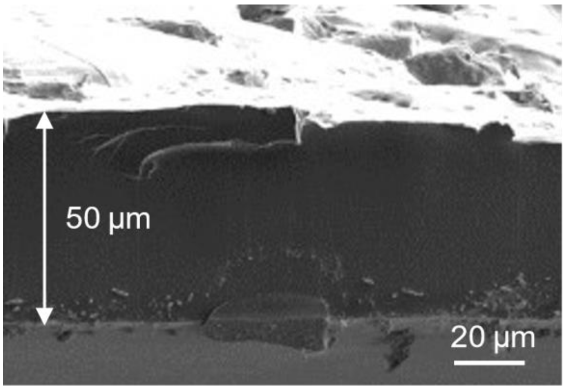

Figure S17. Cross-sectional SEM image of GPE.

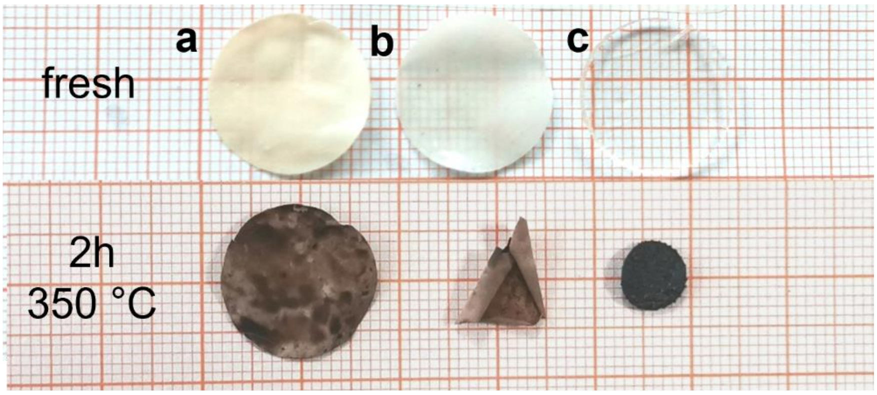

Figure S18. Photograph shows the thermal stability of FMPE (a), MPE (b) and GPE (c) after exposure to 350 °C for 2 h.

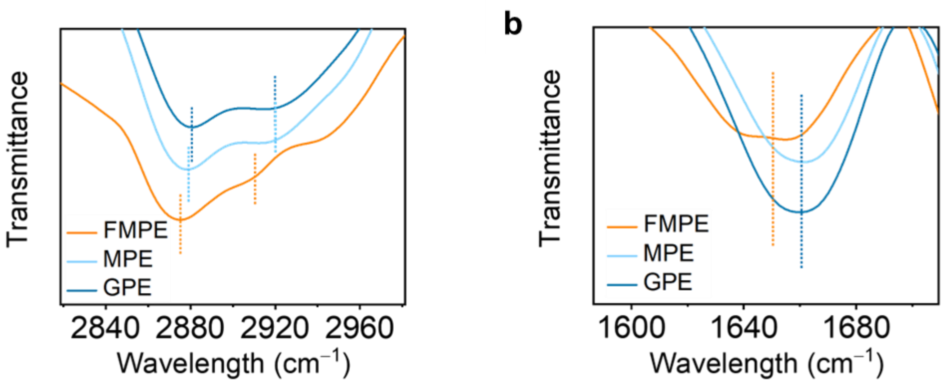

Figure S19. FTIR spectra of FMPE, MPE and GPE.

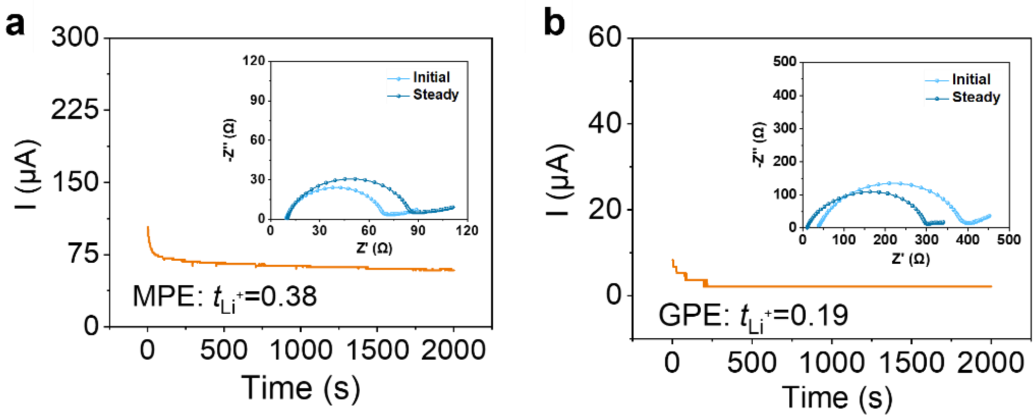

Figure S20. The chronoamperometry profiles of Li||Li symmetrical cells using MPE (a) and GPE (b) under a polarization voltage of 10 mV and the EIS before and after the polarization.

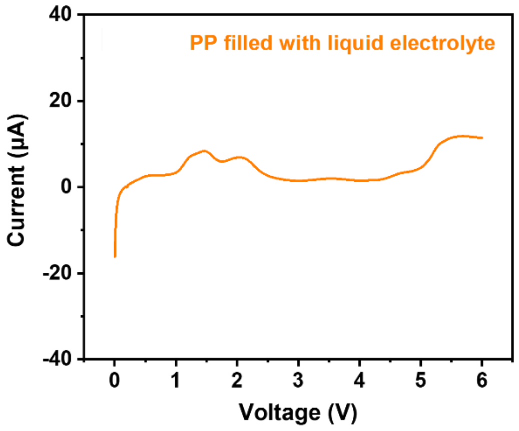

Figure S21. Electrochemical stability window of PP filled with liquid electrolyte determined by LSV.

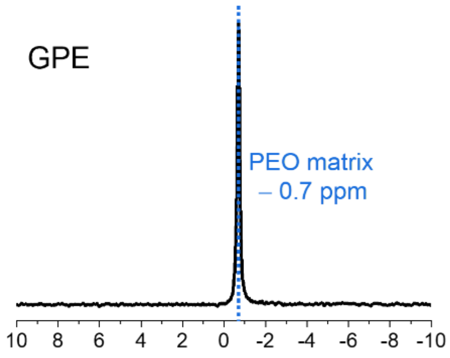

Figure S22. ^{6}Li spectra of the GPE.

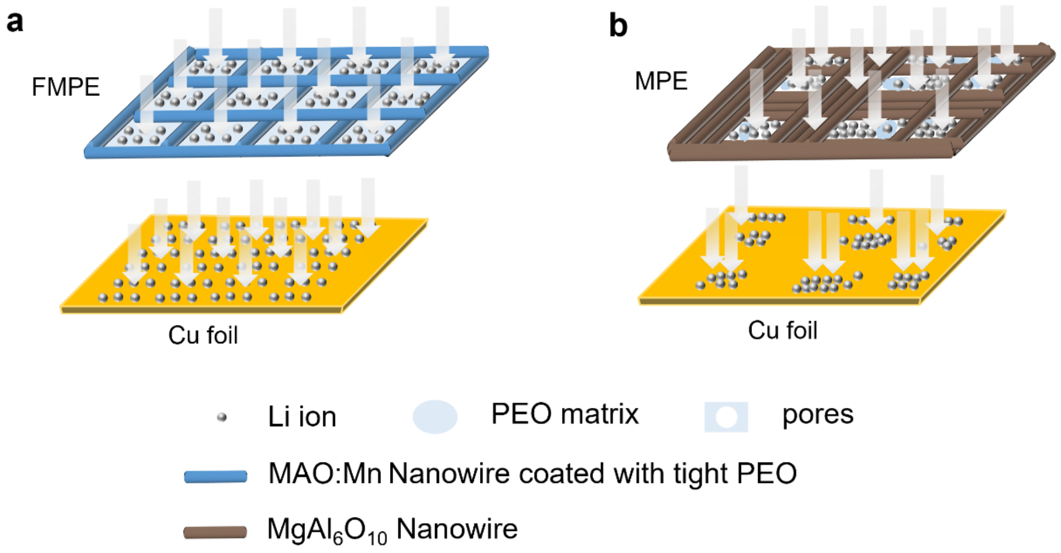

Figure S23. The Schematic diagram of Li ion deposition on Cu foils using patterning FMPE (a) and patterning MPE (b).

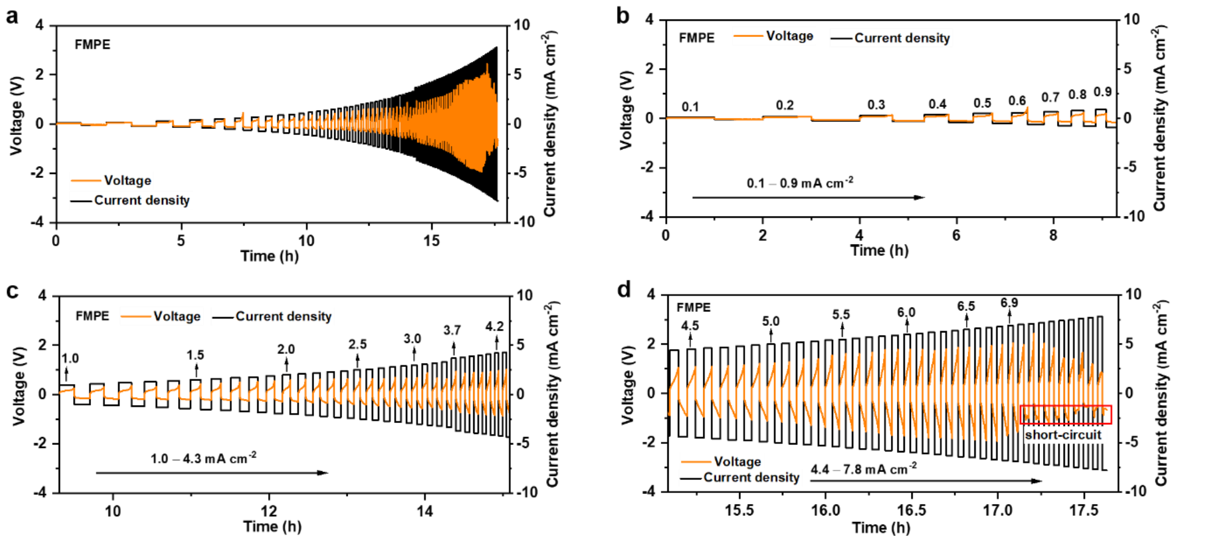

Figure S24. Galvanostatic cycling curves at step-increased current densities of Li||Li symmetric cells assembled with FMPE.

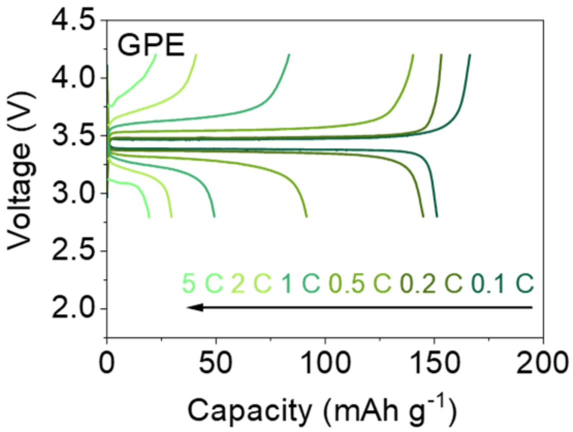

Figure S25. Charge/discharge curves of Li|GPE|LFP cell from 0.1 to 5 C.

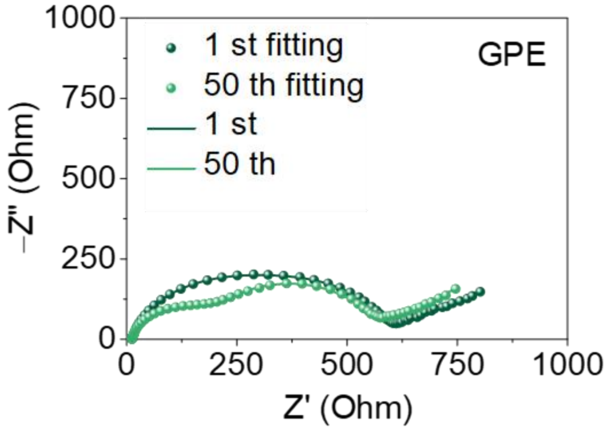

Figure S26. EIS plots of Li|GPE|LFP cell after different cycles.

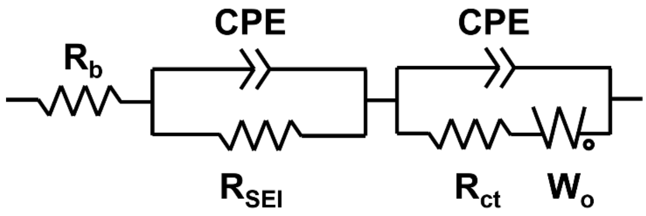

Figure S27. Equivalent circuit model used for the EIS simulation.

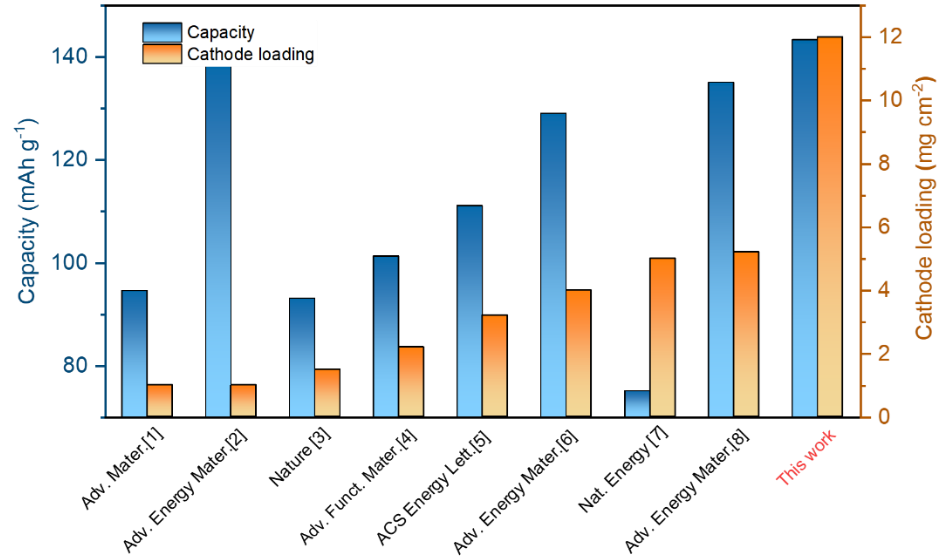

Figure S28. Comparison of the electrochemical performances of selected typical quasi-solid-state electrolytes.

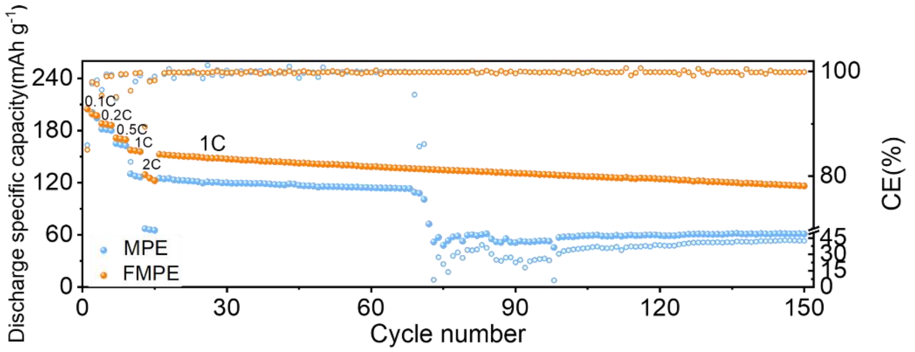

Figure S29. Cycling performances of Li||NCM811 cells using FMPE and MPE under 30 °C.

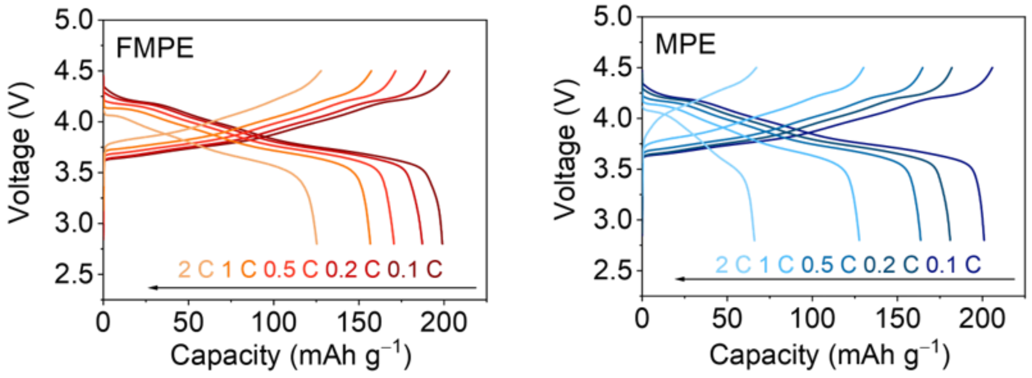

Figure S30. Charge/discharge curves of Li||NCM811 cells with FMPE and MPE from 0.1 to 2 C.

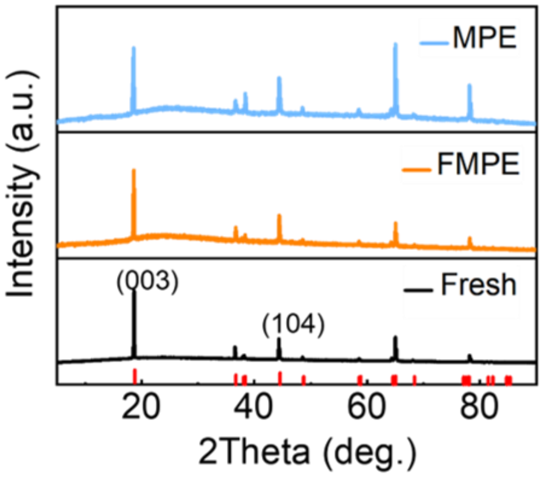

Figure S31. XRD of NCM811 cathodes before and after 70 cycles in FMPE and MPE.

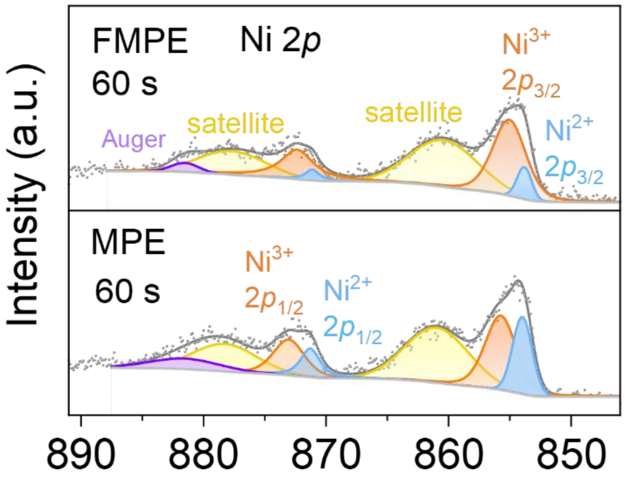

Figure S32. XPS spectra of Ni 2p spectrum of NCM811 cathodes in FMPE and MPE.

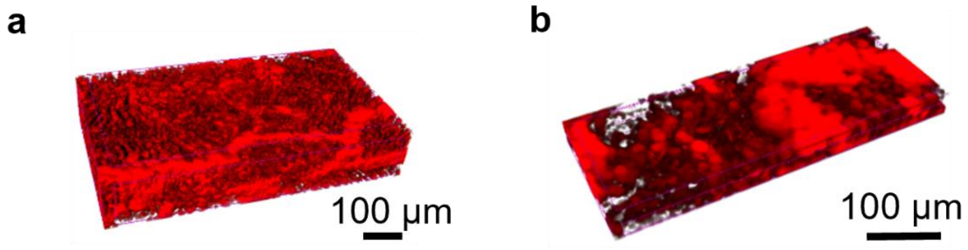

Figure S33. 3D maps and diameter distribution of the pores in dead Li layer using MPE (a) and FMPE (b).

Table S1. The amount of liquid electrolyte uptake of different membranes.

Membranes	Before LE uptake (mg)	After LE uptake (mg)	Liquid electrolyte uptake (%)
**GPE**	12.9	25.0	93.8
**MPE**	28.0	39.5	41.1
**FMPE**	25.3	36.5	44.3

Table S2. Simulation results of Li|FMPE|LFP，Li|MPE|LFP and Li|GPE|LFP at different cycles.

	FMPE		MPE		GPE	
	R_{ct}/Ω	R_{SEI}/Ω	R_{ct}/Ω	R_{SEI}/Ω	R_{ct}/Ω	R_{SEI}/Ω
**1st**	76.86	132.5	167.1	59.84	166.6	374.9
**50th**	72.08	122.8	198.4	116.4	322.9	198.1

Table S3. Comparation of FMPE electrolyte with other reported solid-state electrolytes.

State	Filler	Polymer/lithium salt	σ (S cm^{-1})	t_{Li^+}	Electrochemical stability window (V)	Li-Li battery	Full battery performance	cathode loading (mg cm^{-2})	Ref
Quasi-Solid-State	$MgAl_2O_4:Mn^{4+}$	PEO/$LiPF_6$	5.66×10^{-4} at 25 °C	0.38	5.2	0.2 mA cm^{-2} (0.2 mAh cm^{-2}) for 560 h at 30 °C	Li||LFP, 1 C, 144.1 mAh g^{-1}, 90% capacity retention after 500 cycles, 30 °C	2.4	This work
							Li||LFP, 0.1 C-0.3 C, 143.3 mAh g^{-1}, 88% capacity retention after 120 cycles, 30 °C	12	
	MMT	ETPTA/PVDF-HFP/$LiPF_6$	1.60×10^{-3} at 25 °C	0.78	5	0.5 mA cm^{-2} (0.25 mAh cm^{-2}) for 500 h at 25 °C	Li||LCO, 0.5 C, 135 mAh g^{-1}, 96% capacity retention after 200 cycles, 25 °C	5.2	Adv. Energy Mater., 2020, 10, 2003114
	LATP	PolyILs/LiTFSI	1.7×10^{-4} at 50 °C	——	4.9	0.05 mA cm^{-2} (0.05 mAh cm^{-2}) for 3500 h at 50 °C	Li||LFP, 0.1 C, 159.5 mAh g^{-1}, 99.6% capacity retention after 100 cycles, 50 °C	2.5-3.0	Adv. Funct. Mater., 2022, 32, 2108706

**Quasi-Solid-State**	SiO_2 aerogel	PEO/LiTFSI	6.00×10^{-4} at 30 °C	0.38	4.4	0.05 mA cm^{-2} (0.05 mAh cm^{-2}) for 450 h at 18 °C	Li‖LFP, 0.5 C, 94.5 mAh g^{-1}, 84% capacity retention after 200 cycles, 18 °C	1	Adv. Mater., 2018, 30, 1802661
							Li‖LFP, 0.2 mA cm^{-2}, 146.7 mAh g^{-1}, 95% capacity retention after 13 cycles, 55 °C	7.4	
							Li‖LFP, 0.2 mA cm^{-2}, 148.65 mAh g^{-1}, 100% capacity retention after 25 cycles, 55 °C	13.6	
	——	BA/PEGDA/LiTFSI	1.1×10^{-3} at 20 °C	0.75	4.75	10 mA cm^{-2} (10 mAh cm^{-2}) for 1500 h at 20 °C	Li‖LFP, 1 C, 93 mAh g^{-1}, 95% capacity retention after 1000 cycles, 20 °C	1.5	Nature, 2022, 601, 217-222
	ZIF-4 glass	—/LiTFSI	1.61×10^{-4} at 30 °C	0.88	4	0.1 mA cm^{-2} (0.1 mAh cm^{-2}) for 500 h at 30 °C	Li‖LFP, 1 C, 101.2 mAh g^{-1}, 100% capacity retention after 500 cycles, 30 °C	2.2	Adv. Funct. Mater., 2021, 31, 2104300

	LLZTO	PEO/LiTFSI	1.43×10^{-3} at 25 °C	——	4.8	0.2 mA cm^{-2} (0.1 mAh cm^{-2}) for 500 h at 25 °C	Li‖LFP, 1 C, 138.2 mAh g^{-1}, 90% capacity retention after 1000 cycles, 25 °C	——	Angew. Chem. Int. Ed., 2021, 60, 12116-12123
**Quasi-Solid-State**	——	BC-g-PLiSTFSI-b-PEGM	3.1×10^{-4} at RT	0.85	——	1 mA cm^{-2} (1 mAh cm^{-2}) for 3300 h at 30 °C	Li‖LFP, 1 C, 99 mAh g^{-1}, 77% capacity retention after 300 cycles, 30 °C	——	Adv. Mater., 2020, 2100943
	——	PolyDOL/LiTFSI	1×10^{-3} at RT	——	——	1 mA cm^{-2} (1 mAh cm^{-2}) for 200 h at RT	Li‖LFP, 1 C, 75 mAh g^{-1}, 83% capacity retention after 700 cycles, RT	5	Nat. Energy, 2019, 4, 365-373
	——	MIC/LiFSI	1×10^{-3} at 25 °C	0.6	5.6	0.2 mA cm^{-2} (0.1 mAh cm^{-2}) for 2000 h at RT	——	——	Nat. Mater., 2021, 20, 1255-1263

**Quasi-Solid-State**	SiO_2	TPGDA/$LiFP_6$	1.95×10^{-3} at 25 °C	0.45	4.9	1 mA cm^{-2} (1 mAh cm^{-2}) for 400 h at 25 °C	Li||LFP, 1 C, 125.5 mAh g^{-1}, 88.42% capacity retention after 700 cycles, 25 °C	——	ACS Energy Lett., 2020, 5, 1681-1688
	hBN	Ionogel/LiFSI	1.6×10^{-3} at 25 °C	——	5	——	Li||LFP, 1 C, 111 mAh g^{-1}, 78% capacity retention after 500 cycles, RT	3.2	ACS Energy Lett., 2022, 7, 1558-1565
	SiO_2	PDMA/$LiFP_6$	1.2×10^{-4} at RT	0.43	4.45	0.05 mA cm^{-2} (0.1 mAh cm^{-2}) for 430 h at RT	Li||LFP, 1 C, 138.8 mAh g^{-1}, 95.2% capacity retention after 500 cycles, RT	1	Adv. Energy Mater., 2019, 9, 1900257
	hBN	Ionogel/LiFSI	1×10^{-3} at RT	——	5.3	——	Li||LFP, 10 C, 144 mAh g^{-1}, 90% capacity retention after 100 cycles, 175 °C	2	ACS Nano, 2019, 13, 9664-9672

	——	COF-gel/LiTFSI	——	——	——	——	Li||LFP, 0.5 C, 151.7 mAh g^{-1}, 95.83% capacity retention after 759 cycles, 25 °C	3	Angew. Chem. Int. Ed., 2021, 60, 12116-12123
**Quasi-Solid-State**	ZIF-8/Al_2O_3	PVDF-HFP/$LiPF_6$	1.63×10^{-3} at RT	0.74	5	0.2 mA cm^{-2} (1 mAh cm^{-2}) for 1000 h at 25 °C	Li||LLO, 0.5 C, 216 mAh g^{-1}, 84.6% capacity retention after 500 cycles, 25 °C	2.5	ACS Energy Lett., 2022, 7, 42-52
	——	TTE/LiTFSI	9.6×10^{-4} at RT	——	5	0.5 mA cm^{-2} (0.5 mAh cm^{-2}) for 560 h at RT	Li||LFP, 0.2 C, 129 mAh g^{-1}, 88% capacity retention after 120 cycles, 25 °C	3.5-4	Adv. Energy Mater., 2020, 10, 1903325

	Ca-CeO_2	PEO/LiTFSI	1.30×10^{-4} at 60 °C	0.453	4.1	0.1 mA cm^{-2} (0.05 mAh cm^{-2}) for 1000 h at 60 °C	Li‖LFP, 1 C, 93 mAh g^{-1}, 74.4% capacity retention after 200 cycles, 60 °C	——	Adv. Energy Mater., 2020, 10, 2000049
	$Li_{21}Si_5$	PEO/LiTFSI	3.90×10^{-5} at 30 °C	0.43	5.1	0.2 mA cm^{-2} (0.1 mAh cm^{-2}) for 300 h at 45 °C	Li‖LFP, 0.5 C, 113 mAh g^{-1}, 79.5% capacity retention after 200 cycles, 45 °C	1.19	Adv. Mater., 2021, 33, 2004711
**All-Solid-State**	LLZTO	PEO/LiTFSI	2.30×10^{-5} at 30 °C	——	5.03	0.2 mA cm^{-2} (0.1 mAh cm^{-2}) for 400 h at 30 °C	Li‖LFP, 0.1 C, 97.7 mAh g^{-1}, 82.4% capacity retention after 200 cycles, 30 °C	1.5	Adv. Energy Mater., 2019, 9, 1804004
	LGPS	PEO/LiTFSI	9.83×10^{-4} at 25 °C	0.68	5.1	2 mA cm^{-2} (4 mAh cm^{-2}) for 6700 h at 25 °C	Li‖LFP, 0.5 C, 149 mAh g^{-1}, 91% capacity retention after 150 cycles, 60 °C	——	Adv. Mater., 2020, 32, 2000399

	Cd0.1Ce0.9O 1.95	PEO/LiTFSI	1.90×10^{-4} at 30 °C	0.26	4	0.1 mA cm^{-2} (0.05 mAh cm^{-2}) for 800 h at 35 °C	Li‖LFP, 0.1 mA cm^{-2}, 128 mAh g^{-1}, 80% capacity retention after 150 cycles, 35 °C	3-5	Angew. Chem. Int. Ed., 2020, 59, 4131-4137
	LATP	P(PEGMEA)/$LiPF_6$	2.00×10^{-4} at 25 °C	0.48	4.5	0.1 mA cm^{-2} (0.1 mAh cm^{-2}) for 500 h at 25 °C	Li‖LCO, 0.1 C, 117 mAh g^{-1}, 82% capacity retention after 120 cycles, 45 °C	1.46	Adv. Sci., 2021, 8, 2003887
**All-Solid-State**	MOFs (UIO-66)	PETMP/PEGDA/ LiTFSI	2.26×10^{-4} at 30 °C	0.44	5.4	0.1 mA cm^{-2} (0.05 mAh cm^{-2}) for 1350 h	Li‖LFP, 0.5 C, 123 mAh g^{-1}, 85.6% capacity retention after 500 cycles, 40 °C	2.4	Adv. Mater., 2020, 32, 2001259
	LLZTO@PAN	PEO/LiTFSI	1.10×10^{-4} at 60 °C	0.66 (60 °C)	4.35	0.1 mA cm^{-2} (0.1 mAh cm^{-2}) for 300 h at 60 °C	Li‖LFP, 0.1 C, 149.6 mAh g^{-1}, 89.6% capacity retention after 100 cycles, 45 °C	——	J. Am. Chem. Soc., 2021,143, 5717-5726

All-Solid-State	LLTO	PAN/$LiClO_4$	6.05×10^{-5} at 30 °C	0.42	——	——	——	——	Nat. Energy, 2017, 2, 17035
	PI	PEO/LiTFSI	2.30×10^{-4} at 30 °C	——	——	0.1 mA cm^{-2} (0.05 mAh cm^{-2}) for 1000 h at 60 °C	Li‖LFP, 0.5 C, 100 mAh g^{-1}, 88.5% capacity retention after 300 cycles, 60 °C	1.5	Nat. Nanotechnol., 2019, 14, 705-711
	Vermiculite sheets	PEO/LiTFSI	1.89×10^{-4} at 25 °C	0.5	——	1 mA cm^{-2} (0.17 mAh cm^{-2}) for 1300 h at 35 °C	Li‖LFP, 0.5 C, 110 mAh g^{-1}, 82% capacity retention after 200 cycles, 35 °C	0.8	Adv. Funct. Mater., 2019, 29, 1900648
	S-hBN	PEO/TEGDME/LiTFSI	0.47×10^{-3} at 25 °C	——	——	——	Li‖LFP, 0.1 C, 132 mAh g^{-1}, 91% capacity retention after 100 cycles, 25 °C	2.3	Adv. Funct. Mater., 2021, 31, 2006683

All-Solid-State	PE	PEO/LiTFSI	3.68×10^{-5} at 30 °C	——	——	0.1 mA cm^{-2} (0.1 mAh cm^{-2}) for 1500 h at 60 °C	Li‖LFP, 1 C, 96 mAh g^{-1}, 66% capacity retention after 500 cycles, 60 °C	1.5	Adv. Energy Mater., 2019, 9, 1902767
							Li‖LFP, 0.1 C, 127 mAh g^{-1}, 94.4% capacity retention after 50 cycles, 60 °C	7	
	LZP	PEO/LiTFSI	1.20×10^{-4} at 30 °C	0.36	4.7	0.1 mA cm^{-2} (0.05 mAh cm^{-2}) for 100 h at 40 °C	Li‖LFP, 0.2 C, 120 mAh g^{-1}, 77.4% capacity retention after 100 cycles, 40 °C	3	J. Am. Chem. Soc., 2020,142, 2497-2520
	LLTO	PEO/LiTFSI	2×10^{-5} at 25 °C	——	——	0.05 mA cm^{-2} (0.05 mAh cm^{-2}) for 400 h at 65 °C	Li‖LFP, 0.1 C, 120 mAh g^{-1}, 82.6% capacity retention after 50 cycles, 65 °C	2	Adv. Mater., 2019, 32, 1906221

	LLZO	PEO/LiTFSI	2.39×10^{-4} at 25 °C	——	5.5	1 mA cm^{-2} (1 mAh cm^{-2}) for 325 h at 60 °C	Li||LFP, 0.5 C, 154.7 mAh g^{-1}, 97.4% capacity retention after 70 cycles, 60 °C	1.68	Adv. Funct. Mater., 2019, 29, 1805301
**All-Solid-State**	Al-LLZTO	PVDF-HFP/LiTFSI	3.75×10^{-5} at 30 °C	0.45	5.1	0.1 mA cm^{-2} (0.03 mAh cm^{-2}) for 1000 h at 30 °C	Li||LFP,1 C, 134 mAh g^{-1}, 92.4% capacity retention after 140 cycles, 30 °C	——	Adv. Funct. Mater., 2022, 32, 2111919
	LAGP	PEGMA/LiTFSI	2.37×10^{-4} at 30 °C	0.87	——	0.1 mA cm^{-2} (0.1 mAh cm^{-2}) for 3500 h at 30 °C	Li||LFP, 0.5 C, 122 mAh g^{-1}, 81% capacity retention after 500 cycles, 60 °C	1	Adv. Energy Mater., 2022, 2200368

Supporting References

[1] D. Lin, P. Y. Yuen, Y. Liu, W. Liu, N. Liu, R. H. Dauskardt, Y. Cui, *Adv. Mater.* **2018**, *30,* 1802661.

[2] L. Yu, S. Guo, Y. Lu, Y. Li, X. Lan, D. Wu, R. Li, S. Wu, X. Hu, *Adv. Energy Mater.* **2019**, *9,* 1900257.

[3] M. J. Lee, J. Han, K. Lee, Y. J. Lee, B. G. Kim, K.-N. Jung, B. J. Kim, S. W. Lee, *Nature* **2022**, *601,* 217.

[4] G. Jiang, C. Qu, F. Xu, E. Zhang, Q. Lu, X. Cai, S. Hausdorf, H. Wang, S. Kaskel, *Adv. Funct. Mater.* **2021**, *31,* 2104300.

[5] C. M. Thomas, W. J. Hyun, H. C. Huang, D. Zeng, M. C. Hersam, *ACS Energy Lett.* **2022**, *7,* 1558.

[6] Y. Zhang, Y. Shi, X. C. Hu, W. P. Wang, R. Wen, S. Xin, Y. G. Guo, *Adv. Energy Mater.* **2020**, *10,* 1903325.

[7] Q. Zhao, X. Liu, S. Stalin, K. Khan, L. A. Archer, *Nature Energy* **2019**, *4,* 365.

[8] Y. M. Jeon, S. Kim, M. Lee, W. B. Lee, J. H. Park, *Adv. Energy Mater.* **2020**, *10,* 2003114.
